# Nanopore sequencing of brain-derived full-length circRNAs reveals circRNA-specific exon usage, intron retention and microexons

**DOI:** 10.1038/s41467-021-24975-z

**Published:** 2021-08-10

**Authors:** Karim Rahimi, Morten T. Venø, Daniel M. Dupont, Jørgen Kjems

**Affiliations:** 1 Department of Molecular Biology and Genetics (MBG), Aarhus University, Aarhus, Denmark; 2 Interdisciplinary Nanoscience Center (iNANO), Aarhus University, Aarhus, Denmark; 3 Omiics ApS, Aarhus, Denmark

**Keywords:** Sequencing, RNA splicing, Non-coding RNAs

## Abstract

Circular RNA (circRNA) is a class of covalently joined non-coding RNAs with functional roles in a wide variety of cellular processes. Their composition shows extensive overlap with exons found in linear mRNAs making it difficult to delineate their composition using short-read RNA sequencing, particularly for long and multi-exonic circRNAs. Here, we use long-read nanopore sequencing of nicked circRNAs (circNick-LRS) and characterize a total of 18,266 and 39,623 circRNAs in human and mouse brain, respectively. We further develop an approach for targeted long-read sequencing of a panel of circRNAs (circPanel-LRS), eliminating the need for prior circRNA enrichment and find >30 circRNA isoforms on average per targeted locus. Our data show that circRNAs exhibit a large number of splicing events such as novel exons, intron retention and microexons that preferentially occur in circRNAs. We propose that altered exon usage in circRNAs may reflect resistance to nonsense-mediated decay in the absence of translation.

## Abstract (web summary)

Short-read sequencing methods cannot delineate internal exon composition and alternative splicing events of long and multi-exon circular RNAs (circRNAs). Here the authors provide a global map of full-length circRNAs by long-read sequencing in human and mouse brain samples.

## Introduction

Circular RNA (circRNA) constitutes an abundant and partially conserved group of RNAs derived from both coding and non-coding linear transcripts. They are the outcome of a unique splicing event in which the 5′ splice donor back-splices to the upstream 3′ splice acceptor, resulting in a closed circular RNA. The formation of a unique back-splice junction (BSJ) differentiates the circRNAs from their linear counterpart. CircRNAs are highly expressed in the central nervous system where many of them are regulated during development^1–3^, inflammation^4^, and also found to function as biomarkers in cancer^5–7^. Only a limited number of circRNAs have been characterized functionally. The most extensively studied circRNA, ciRS-7/CDR1-AS, appears to act as a regulator of microRNA miR-7^8–10^ and its removal in mouse brain causes cognitive changes^11^.

High-throughput techniques, including Illumina-based RNA-seq^12^, microarrays^13,14^, and NanoString^15^ have been employed to profile circRNA expression by detecting and counting the number of unique BSJ sequences. However, none of the short-read sequencing techniques have so far been able to elucidate the exon composition of circRNAs longer than 400–500 nucleotides (nts) in the background of linear mRNA. A few studies have addressed this issue using computational analysis of paired-end reads but this has also been limited to small circRNAs^16,17^.

The availability of long-read sequencing techniques such as Oxford Nanopore Technology (ONT)^18^ and Single-Molecule Real-Time^19^ from Pacific Biosciences (PacBio) has opened up a new era in genomic and transcriptomic studies by allowing ultra-long sequencing reads^20–23^. ONT has been used to sequence poly(A)-purified RNA and reported a significant number of trans-spliced RNAs^24^; however, the inclusion of a poly(A) selection step prevented the detection of circRNAs. Another study combined nanopore sequencing with a PCR-based approach by using end-to-end divergent primers to create BSJ reads and find different isoforms of circNPM1; however, this study was limited to this single circRNA^25^.

Here, we applied for the first time the ONT platform to provide a deep characterization of alternative splicing events in circRNAs at a global scale (see the first version of this study on bioRxiv)^26^. We find that mRNA and circRNA exhibit divergent exon composition with a significant number of circRNA-specific exons and splicing events seen in human and mouse brain. Strikingly, many of the so-called microexons, reported to function in development, behavior, and autism spectrum disorder, reviewed by Gonatopoulos-Pournatzis & Blencowe^27^, appear to be preferentially found in circRNAs. It raises the possibility that circRNA-specific RNA elements may exert novel functions which are absent in the linear transcriptome.

## Results

### Nanopore long-read sequencing of global circRNA and poly(A) RNA

**Fig. 1:**
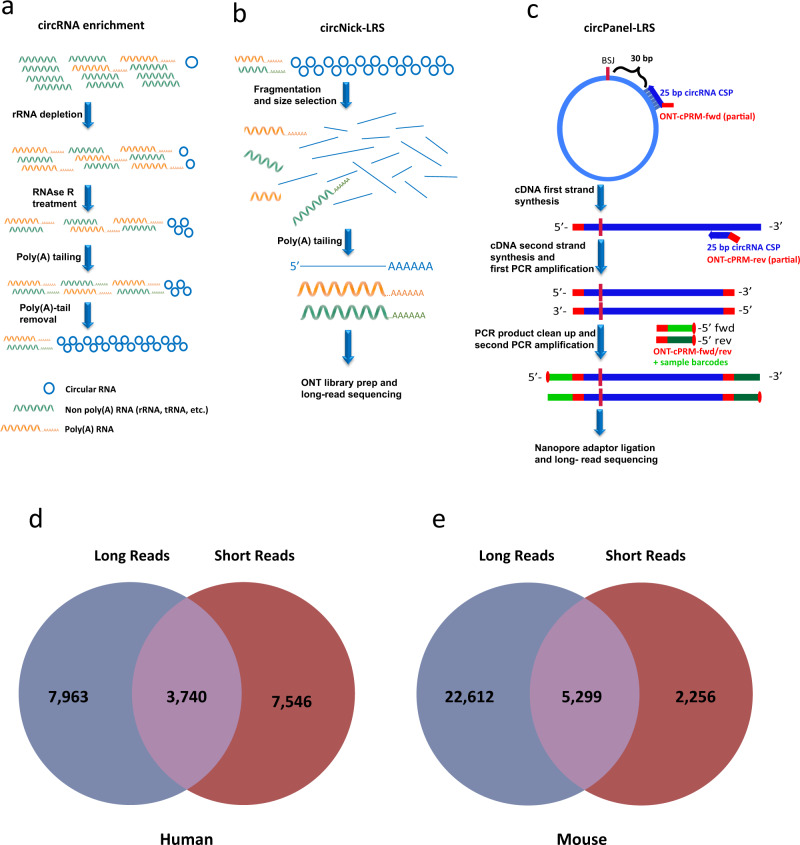
Workflow for circRNA ONT sequencing. **a** CircRNA enrichment protocol. Total human or mouse brain RNA was subjected to DNase treatment and rRNA depletion before treatment with RNase R to digest the linear RNAs. To further enrich for circRNAs, the remaining linear RNAs were polyadenylated and depleted using poly(A) purification beads. **b** CircRNA library preparation. The enriched circRNA pool was nicked using an optimized protocol to generate large fragments. After phosphorylation of the 5′ ends of the linearized RNAs, the obtained RNA pool was subjected to polyadenylation to align with the ONT sequencing protocol. **c** CircRNA panel Long-Read Sequencing (circPanel-LRS). Total RNA was reverse transcribed using a panel of circRNA-specific primers (CSP) extended with an oligo sequence partially overlapping the ONT-cPRM (ONT cDNA primer) forward primer oligo. The second strand of the cDNA was synthesized using a panel of reverse circRNA-specific paired primers extended with an oligo sequence partially overlapping the ONT-cPRM reverse primer oligo. Six PCR cycles were run using the first and second strand cDNA synthesis primer oligos without prior cleanup for the first PCR amplification. After cleaning up the PCR product, the resulting library is amplified using ONT sequencing adjusted and sample indexed primers. Equal molar ratios of the barcoded libraries were mixed and ligated to the ONT sequencing adapter before loading to flowcells for sequencing. **d**, **e** Number of circRNAs detected using long- and short-read sequencing in human and mouse brain tissue.

Only a small fraction of RNA in mammalian cells is circular (~0.1% of the rRNA-depleted RNA pool in the mouse nervous system^28^). To focus our sequencing on this small fraction of RNA, we developed a circRNA enrichment protocol with some resemblance to the RPAD protocol^29^ to deplete both ribosomal and linear RNA. In brief, total RNA from mouse or human brain was treated with RiboZero and RNase R to remove ribosomal and linear RNA, respectively. Since some linear RNAs are resistant to RNase R, we conducted an additional polyadenylation step followed by poly(A)-depletion to remove the remaining linear RNA (see Fig. 1a for an overview of the full enrichment protocol). Quantification of selected circRNAs, linear mRNA, and ribosomal RNA confirmed that this protocol led to a nearly 1000-fold enrichment of circRNAs (Supplementary Fig. 1). The circRNA-enriched pool was nicked by gentle hydrolysis prior to long-read sequencing (circNick-LRS), followed by 3′ end polyadenylation to align with the standard ONT protocol (Fig. 1b).

Nanopore data was collected using MinKnow (v 1.0.1) software. Applying the circNick-LRS protocol to enriched circRNA from human and mouse brain samples yielded 4,273,440 and 12,683,908 high quality filtered sequencing reads, of which 189,862 (4.44%) and 790,634 (6.23%) contained circRNA back-splice junctions (BSJ), respectively, (Table 1). Mapping the reads to the reference genomes yielded a total of 18,266 and 39,623 unique circRNAs in human and mouse brain samples, respectively (Supplementary Data 1). Scatter plots of read length versus average read quality for ONT sequencing are shown in Supplementary Fig. 2.

**Table 1:** Information on reads that map to circRNAs and the BSJ reads in each dataset.

	CircNick-LRS		CircPanel-LRS	
	Human brain	Mouse brain	Human brain	SH-SY5Y cells
Filtered reads	4,273,440	12,683,908	4,005,162	3,954,246
Back splice junction reads	189,862 (4.4% of filtered reads)	790,634 (6.2% of filtered reads)	2,508,109 (62.6% of filtered reads)	2,963,459 (75% of filtered reads)
circRNA mapping reads	619,770 (14.5% of filtered reads)	4,470,533 (35.3% of filtered reads)	3,922,818 (98% of filtered reads)	3,832,609 (97% of filtered reads)
Unique circRNAs	18,266	39,623	403	340
CircBase circRNAs	10,094	7,589	157	136
CircAtlas circRNAs	14,698	27,501	296	223
CIRCpedia circRNAs	12,949	19,466	199	150
Novel circRNAs	2,851	11,380	118	129
Multi round circRNAs	–	–	30,009 reads	45,110 reads

To enable a global comparison to the linear mRNA transcriptome, we performed ONT full-length poly(A) RNA sequencing on the same brain samples. This resulted in a total of 21.7 and 15.4 million reads for human and mouse brain-derived RNA samples, respectively. The FLAIR tool (Full-Length Alternative Isoform analysis of RNA)^30^ was used to analyze the long-read mRNA data and detect linear mRNA isoforms. In total, 42,782 isoforms derived from 9,754 genes were detected in mouse brain and 47,187 isoforms from 9,512 genes in human brain.

### Long- and short-read sequencing protocols identify distinct pools of circRNAs

To investigate method-specific biases in circRNA expression profiles, we also performed conventional short-read sequencing of rRNA-depleted libraries and used a combination of the find_circ and CIRI2 pipelines for circRNA prediction^10,31^. This approach produced 11,286 and 7,555 different circRNAs in human and mouse brain, respectively (detected with >2 reads; Supplementary Data 2). Comparing the circRNA profiles between long- and short-read sequencing, we found that only 3,740 and 5,299 circRNAs were detected by both methods in human and mouse brain, respectively (Fig. 1d, e). This illustrates that the benefits of long-read sequencing are not limited to elucidating internal circRNA composition but also extend to the detection of circRNAs not seen with short-read sequencing technology, at least at the sequencing depth obtained here. Our data show that highly expressed circRNAs are generally detected using both long- and short-read sequencing methods, but that each method also captures many lower expressed circRNAs not detected by the other technique (Supplementary Fig. 3).

**Fig. 2:**
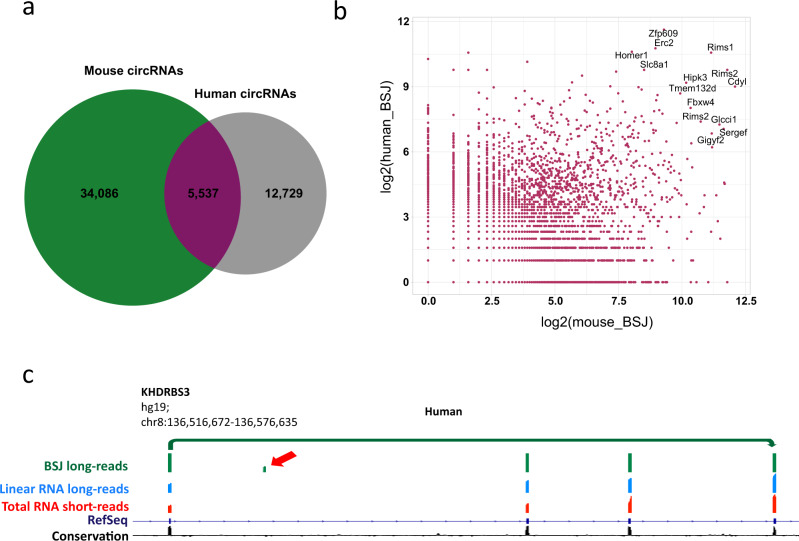
Conservation between human and mouse brain-derived circRNAs. **a** Venn diagram showing the total number of circRNAs found in either human (18,266) or mouse (39,623) or in both (5,537). **b** The number of BSJ-spanning reads detected for mouse (*x*-axis) vs. human (*y*-axis) circRNAs. Only circRNAs that are conserved between human and mouse are included. Some genes like Rims1 generate several different circRNAs. **c** Gene map of human circKHDRBS3, shown as an example where a novel exon (indicated by red arrow) is only detected by ONT sequencing (BSJ long-reads). The green track indicates the BSJ reads in human brain. The light blue track represents reads from full-length linear mRNA sequencing by ONT and the red track indicates short-read sequencing reads for the corresponding sample. The dark blue line refers to RefSeq gene annotation and the black track indicates the level of conservation.

Of the circRNAs detected by circNick-LRS, 5,537 were found in both human and mouse brain, amounting to 30.3% and 14% of the detected human and mouse circRNAs, respectively (Fig. 2a). This is on par with previous studies estimating that 10–20% of expressed circRNAs are conserved between mammalian species^2,32^ and indicates that circNick-LRS is a reliable method to detect true circRNA species.

Among the highest expressed and conserved circRNAs, we found well-known brain circRNAs such as circRims1, circRims2, circHipk3, circCdyl, and circZfp609 using both methods (Fig. 2b). Surprisingly, ciRS-7, previously shown to be highly expressed in human and mouse brain, was missing in the mouse dataset, suggesting that it is unintentionally depleted in the circRNA enrichment protocol, likely due to the presence of internal poly(A) stretches in the circRNA sequence (see discussion). Figure 2c shows the exon map of human-specific circRNA circKHDRBS3 with a side-by-side comparison of sequencing reads generated by short-read, long-read circRNA, and long-read linear poly(A) RNA sequencing. Note that there is an exon exclusively expressed in the circRNA. Interestingly, 2,851 and 11,380 circRNAs from human and mouse, respectively, were not previously annotated in circBase^33^, circAtlas^34^, or CIRCpedia^35^ (marked as novel circRNAs in Table 1 and can be found in Supplementary Data 1).

### Panel sequencing provides high coverage for specific circRNAs

The circNick-LRS protocol provides an unbiased view of global circRNA expression and allowed us to identify a large number of new circRNAs and visualize circRNA structure; however, the read numbers for many circRNAs were still too low to describe a full isoform composition, particularly for lowly expressed circRNAs. To get a more detailed view of the alternative splicing landscape of circRNAs, we developed a library preparation strategy that selectively targets a selected set (panel) of circRNAs followed by Long-Read Sequencing (circPanel-LRS). In this approach circRNA-specific primers for cDNA synthesis are annealed to selected regions either 30 nts downstream of the BSJ or the 5′ end of a shared exon among different known circRNA variants for the targeted locus (Fig. 1c) to selectively amplify all full-length isoforms carrying this BSJ or the conserved targeted exon. Given the use of circRNA-specific primers, no prior circRNA enrichment step is needed and the amount of input RNA sample can be reduced. We noticed that circPanel-LRS has a tendency to produce concatemeric reads, presumably due to repeated rounds of cDNA synthesis on the circular template enabled by strand displacement activity of the reverse transcriptase (Supplementary Fig. 4).

We tested the circPanel-LRS strategy on total RNA purified from human brain and the neuroblastoma SH-SY5Y cell line using primer sets specific for 10 circRNAs chosen based on variable expression levels, sizes and exon numbers (Supplementary Data 3). Nanopore sequencing of the libraries prepared by circPanel-LRS generated 4,005,162 and 3,954,246 high quality filtered reads for human brain and SH-SY5Y samples, respectively, of which 2,508,109 (62.6%) and 2,963,459 (75%) reads crossed the BSJ. In total, we identified 403 and 340 different circRNA variants, respectively, from the 10 chosen loci. Out of these, 118 and 129 were categorized as novel circRNAs (Table 1 and Supplementary Data 4). Supplementary Fig. 5 shows examples of isoforms of circRMST, circHERC1, and circTULP4 detected by circPanel-LRS.

### Multi-exon circRNAs display intron retention

To create a comprehensive map of the circRNA splicing landscape, all mouse and human BSJ-spanning reads were aligned to their respective reference genome. To focus our analysis on significant circRNA-specific-intron retention events, only circRNAs with 20 or more BSJ reads and having a mean intronic mapping percentage above 3% were investigated further. Performing this analysis on the reads obtained using the circNick-LRS protocol we found 1,868 and 4,327 circRNAs in human and mouse, respectively. Of these, 213 and 384 circRNAs contained intron-mapping reads, with 17 and 32 of these containing full-length retained introns in human and mouse, respectively (Supplementary Data 5–6). The circPanel-LRS data showed 25 and 27 circRNA isoforms with intron sequences for human brain and SH-SY5Y cells, respectively, with only one example of conserved intron retention (circRYR2).

**Fig. 3:**
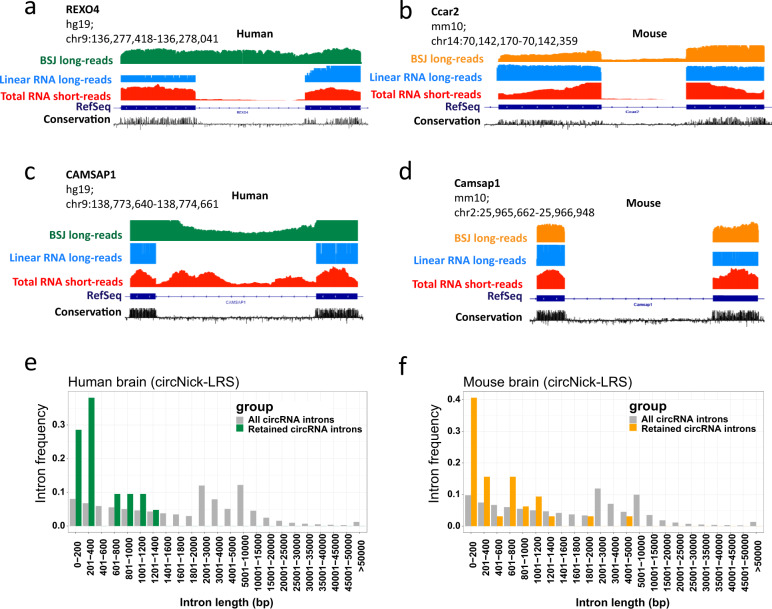
Examples of intron retention events in circRNAs and their frequency and length distribution. **a** Human circREXO4 contains a retained intron not detected in linear mRNA. **b** Mouse CircCcar2 generates different isoforms with retained introns. **c**, **d** CircCAMSAP1 is conserved between human and mouse but only the human circRNA shows intron retention. **e**, **f** The frequency of intron retention in comparison to intron length across all annotated introns in circRNAs genome-wide. Gray bars, frequency of all circRNA introns; green and yellow bars, frequency of retained circRNA introns in human and mouse, respectively. See legend to Fig. 2 for the explanation of tracks.

Some circRNAs detected by circNick-LRS, such as circREXO4 and circCcar2, contain retained introns but are only expressed in either human or mouse (Fig. 3a, b). CircCAMSAP1 is expressed in both human and mouse but intron retention only occurs in human brain (Fig. 3c, d). Comparing all annotated introns in the circRNA genomic regions, we found that shorter introns are more likely to be retained in circRNAs than longer introns (Fig. 3e, f).

### Alternative exon usage prevalence in circRNAs

**Fig. 4:**
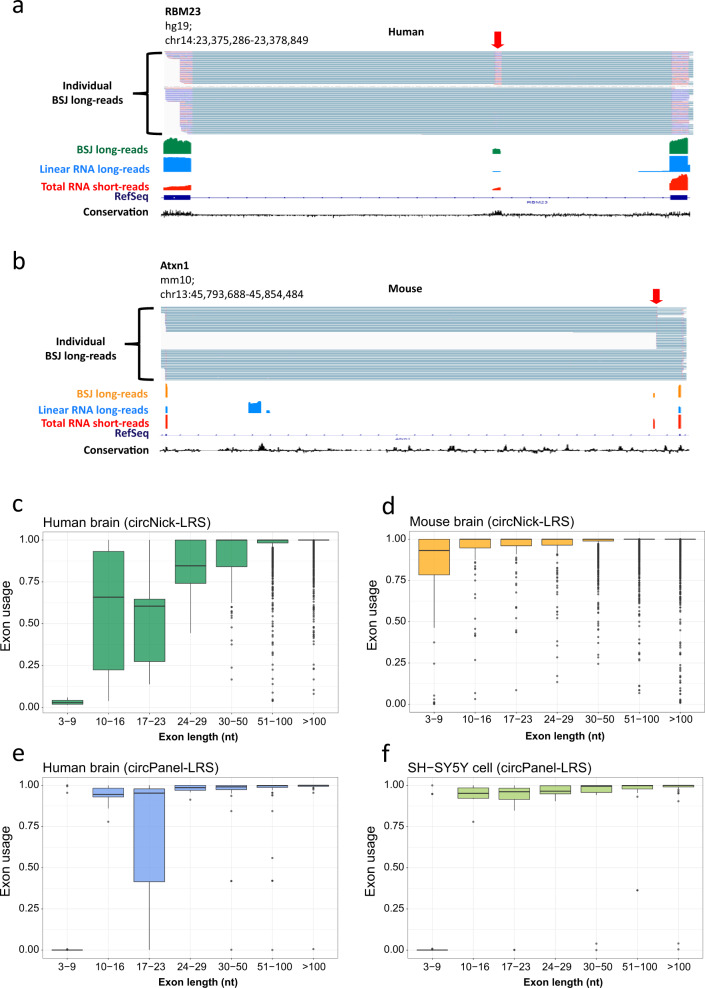
Examples of circRNA-specific alternative splicing. **a** Alternative splicing of circRBM23 (red arrow). The alternative exon is primarily included in circRNA. **b** Example of a circRNA-specific exon in circAtxn1 (red arrow). See legend to Fig. 2 for the explanation of tracks. **c**–**f** The level of exon usage for various exon lengths are shown, ranging from no exon usage, 0 on *y*-scale, to constitutive exons, 1 on *y*-scale. Box plot hinges range from the 25th to the 75th percentile, correspond to the first and third quartiles. Horizontal lines indicate median. The upper whisker extends from the hinge to the largest value, but no further than the 75th percentile + 1.5 × IQR, where IQR is the inter-quartile range, or distance between the first and third quartiles. The lower whisker extends from the hinge to the smallest value, but no further than the 25th percentile − 1.5 × IQR. Dots represent exon lengths outside this boundary. Shown for the samples **c** Human brain (circNick-LRS), **d** Mouse brain (circNick-LRS), **e** Human brain (circPanel-LRS), and **f** SH-SY5Y cells (circPanel-LRS).

The long-read sequencing data also revealed that alternative exon usage is a widespread phenomenon in circRNAs. We found 559 and 681 circRNA isoforms in the circNick-LRS data for human and mouse, respectively, that contained known or novel exons alternatively used in the circRNA and covered by at least 10 reads (Supplementary Data 7). Examples of alternatively spliced circRNA exons are shown for human circRBM23 and mouse circAtxn1 in Fig. 4a, b. Note that in both examples the alternatively used exons are novel and not annotated in RefSeq. The novel exon in human circRBM23 is expressed to a lower extent compared to neighboring exons and the novel exon in mouse circAtxn1 is circRNA-specific and absent in the linear mRNA.

Performing the same analysis for the 10 circRNA loci in the circPanel-LRS dataset revealed 232 and 165 alternatively used exons in human brain and SH-SY5Y samples, respectively, covered by at least 50 reads (Supplementary Data 8). A large number of alternative isoforms were detected from one locus using circPanel-LRS as illustrated for circRIMS1 in the human brain sample (Supplementary Fig. 6). Interestingly, an analysis of the likelihood of exon inclusion revealed that shorter exons are more likely to be alternatively spliced in human and mouse brains (Fig. 4c–f). Our data also confirmed the existence of two previously described human ciRS-7 isoforms of 1485 and 1301 nts caused by optional intron retention^36,37^.

### Detection of novel exons and microexons in circRNA

**Fig. 5:**
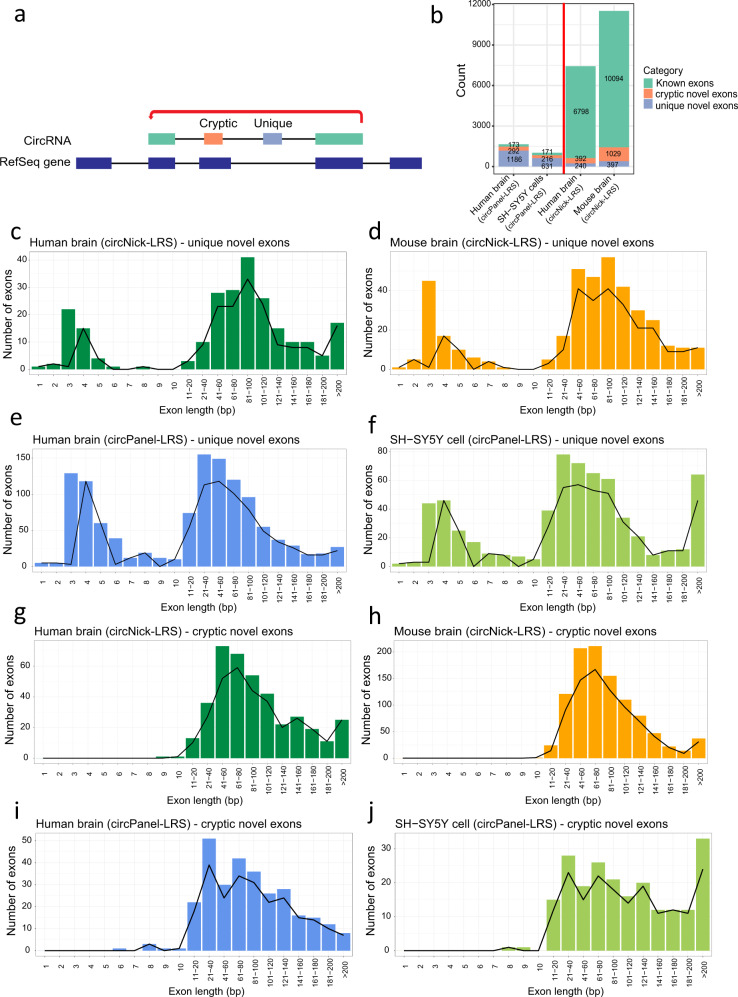
CircRNAs contain unique and cryptic novel exons. **a** Schematic representation of circRNAs with novel exons produced from either cryptic spliced exons (peach) or novel exons (blue gray). The arrow indicates the back-splicing event. **b** Number of known exons, cryptic novel exons, and unique novel exons in circRNAs across all datasets in this study. Only exons covered by at least five long-reads across the back-splice junction are included. **c**–**j** Distribution of exon length of the unique novel (**c**–**f**) and cryptic novel (**g**–**j**) exons in all four datasets made by either circNick-LRS (**c**, **d**, **g**, and **h**) or circPanel-LRS (**e**, **f**, **i**, and **j**).

Approximately 90% of the exons used in circRNAs are annotated in RefSeq or Gencode, but, notably, 8.5% (632) and 12.37% (1,426) of the well-expressed exons (>5 reads) seen in the circNick-LRS data are novel. To further analyze the use of novel exons in circRNAs, we categorized them either as “unique novel exons” (entirely new exons) or “cryptic novel exons” (sharing either the 5′ or 3′ splice sites with the annotated exon; Fig. 5a). Using this annotation, 240 out of 7,430 exons in human and 397 out of 11,520 exons in mouse were detected as unique novel exons and 392 and 1,029 as cryptic novel exons, respectively (all detected with more than 5 reads coverage; Fig. 5b and Supplementary Data 9). CircPanel-LRS, which provides a higher sequencing depth, identified 1,651 and 1,018 exons mapped to the loci of the 10 targeted genes in human brain and SH-SY5Y cells, respectively. Of these, 1,186 and 631 mapped as unique novel exons and 292 and 216 were mapped as cryptic novel exons, respectively (detected with >5 reads coverage; Fig. 5b and Supplementary Data 10). All single-exon circRNAs are excluded from this analysis since complete coverage of detected exons is required within single reads (see Methods section).

**Fig. 6:**
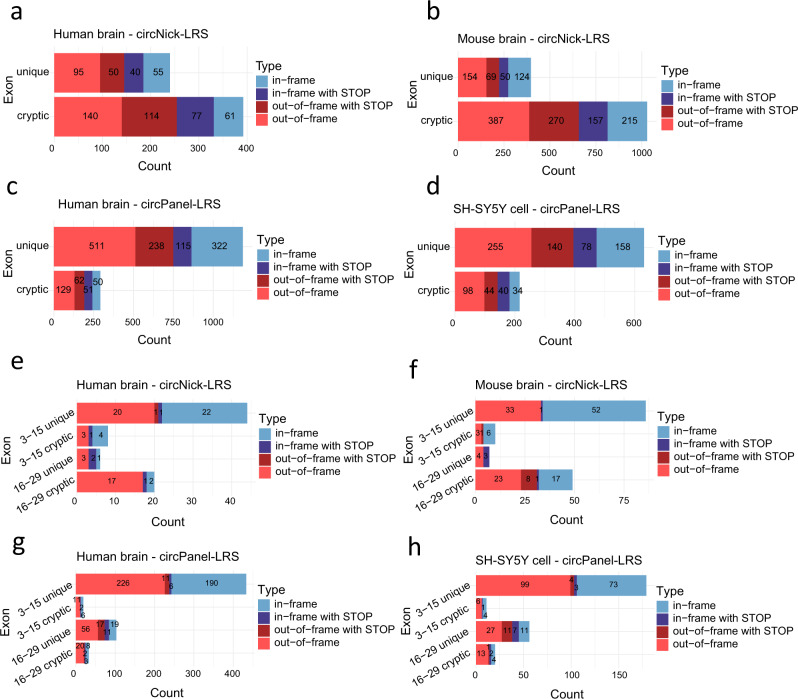
Frequency of frameshifting and stop codons upon inclusion of unique and cryptic novel exons. **a**–**d** Exon frame barplots for all unique novel exons (upper bar) and cryptic novel exons (lower bar) in all four datasets. The type of frameshift caused by the circRNA-specific exons are categorized as in-frame without stop codon (light blue), in frame with stop codon (dark blue), out of frame without stop codon (light red), and out of frame with stop codon (dark red). **e**–**h** Exon frame analysis for all novel microexons (categorized according to length) in all four datasets. All exons are covered by at least 5 long BSJ reads in this analysis.

Most novel circRNA exons found in our dataset are in the 40–200 nts range but a distinct group of very short exons of 3–29 nts were also frequently observed, particularly in the unique novel exons group (Fig. 5c–j). These were categorized as microexons in line with previous findings from linear mRNA. Interestingly, the majority of the novel exons detected in circRNAs resulted in frameshifts or incorporation of stop codons (Fig. 6a–h).

By focusing on the microexons covered by at least 5 BSJ-spanning reads, the circNick-LRS data revealed 53 and 99 unique novel microexons as well as 28 and 59 cryptic novel microexons in human and mouse brain, respectively (Supplementary Data 11 and 12). The circPanel-LRS data, which provides a deeper sequencing coverage for the 10 targeted circRNAs loci, detected 546 and 240 unique novel microexons and 52 and 31 cryptic novel microexons in human brain and SH-SY5Y cells, respectively, with at least 5 BSJ reads (Supplementary Data 13 and 14).

To further analyze the occurrence of well expressed (covered by at least 5 reads) novel microexons in circRNAs, the cryptic- or unique novel exons shorter than 30 nts were divided into two groups (3–15 nts and 16–29 nts). Most unique novel microexons belong to the 3–15 nts group (Fig. 6e, f). CircRNAs are generally not translated and therefore not subjected to nonsense-mediated decay (NMD) if frameshifted. In accordance with this we found ~1/3 in-frame and 2/3 out-of-frame microexons in circRNAs and, within each category, some with at least one stop codon. The circPanel-LRS sequencing showed an even higher number of microexons in the targeted circRNAs, although this may be partly explained by an increased sequencing depth (Fig. 6g, h). A list of all microexons detected in circRNA and covered by >10 reads in circNick-LRS dataset and >50 reads in circPanel-LRS are provided as Supplementary Data 15.

**Fig. 7:**
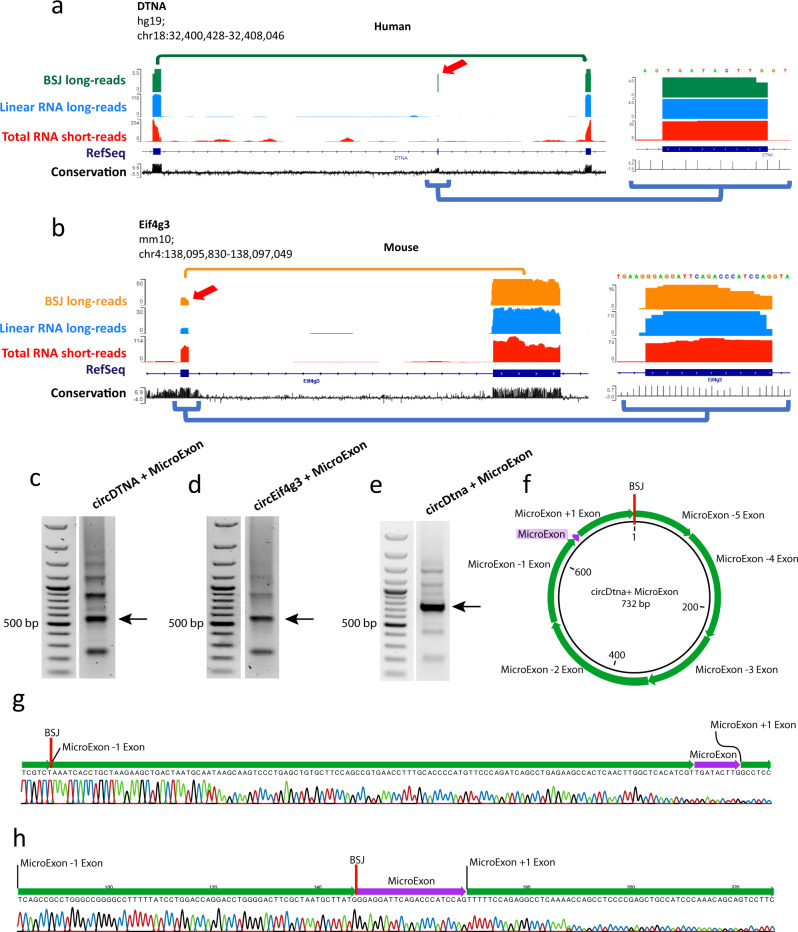
Examples of microexons in circRNAs detected in BSJ-spanning long-reads. **a** Microexon located in circDTNA from human brain detected in 4 out of 5 BSJ-spanning reads (80%; Microexon enlarged to the right). In the long-read linear mRNA, only 4 out of 116 reads (3.5%) include the microexon with the neighboring exons. This microexon is included in 33 out of 204 reads (16%) based on short-read sequencing. **b** Microexon located in circEif4g3 from mouse brain detected in 16 out of 50 BSJ-spanning long-reads (32%) and 7 out of 30 reads (23%) in long-read linear mRNA. Short-read sequencing find the microexon included in 74 out of 114 reads (65%). **c**–**e** RT-PCR validation of circRNAs circDTNA (shown in panel **a**), circEif4g3 (shown in panel **b**), and circDtna (mouse) using back-to-back primers targeting the exons neighboring the microexons and covering the BSJ. Different bands indicate several circRNA isoforms obtained from the same genomic regions and sharing the same targeted exon. Each RT-PCR has been repeated twice and resulted in the same PCR products. **f** Full structure determination of circDtna including the microexon using Sanger sequencing. The circRNA amplicon was gel purified from the PCR band indicated by an arrow in panel **e**. **g**, **h** Microexons in the RT-PCR products (marked by arrows in panels **c**, **d** for circDTNA and circEif4g3 respectively) were validated by Sanger sequencing; the individual sequencing reads cover both BSJ and the microexon.

We noticed that several of the microexons that are preferentially found in circRNA rather than linear RNA are known for their involvement in neuronal dysregulation^38,39^. In particular, microexons in *DTNA* (Fig. 7a) and *Eif4g3* (Fig. 7b) are mainly found in the circRNA form with 80% and 32% inclusion compared to the neighboring exons, but with 3.5% and 23% inclusion in the corresponding linear RNAs, respectively. The circRNA-enriched microexons were validated by RT-PCR for circDTNA (human, Fig. 7c), circEif4g3 (mouse, Fig. 7d) and circDtna (mouse, Fig. 7e) and confirmed by Sanger sequencing (Fig. 7f–h). Other microexons, such as those found in *Ank2*, *Ap1s2*, *Agrn*, *Apbb1*, and *Eif4g3* were found in both circular and linear RNA from the same loci. In the mouse brain, the *Asxl3* and *Patj* genes contain a microexon preferentially included in their respective circular products, while *Fndc3b* contains a microexon exclusively used in circFndc3b.

In human brain, *ERC2*, *APBB1*, and *ASAP1* show microexon inclusion in both circRNA and linear RNA, while *EIF4G3* and *ADGRL3* contain microexons mainly found in the circRNA. In addition, *TMEM245* and *MSANTD3* express microexons that are circRNA-specific and not seen in the linear RNA produced from the same loci.

We compared all microexons in the circNick-LRS dataset (>10 BSJ-spanning reads) with the microexons reported by Irimia et al.^38^ as a reference for neuron-specific microexons. For the human brain sample, 31 out of 181 detected microexons and in mouse sample, 19 out of 499 microexons were found in the reference list (Supplementary Data 16).

## Discussion

Delineation of the exact exon composition of mammalian RNAs is often neglected due to predominant use of short-read sequencing. The invention of long-read sequencing provides a unique opportunity to address the question. In this study, we apply ONT long-read sequencing technology to describe the exon composition of full-length circRNAs as well as the corresponding mRNA. This approach circumvents the limitation of second-generation paired-end sequencing methods where the read length is limited to ~200 nucleotides adjacent to the BSJ, insufficient to read through most circRNAs. Previous studies have presented algorithms to predict circRNA structure and alternative splicing events based on shorter sequencing reads^16,17^; however, neither the current second-generation sequencing protocol nor prediction algorithms can provide a direct view of the full-length sequence of circRNAs.

A limitation of the ONT sequencing method is the relatively low read number (5–10 million reads compared to 1–2 billion reads for short-read sequencing) and we therefore implemented a circRNA enrichment protocol using successive poly(A)-tailing-poly(A) removal steps. However, one should bear in mind that extensive purification can introduce biases in the circRNA pool. For instance, some linear RNAs are known to be resistant to RNase R while some circRNAs are sensitive, presumably because they are nicked during purification. In addition, the probes used for rRNA depletion may have coincidental complementarity to circRNAs and lead to their selective removal. Indeed, we found that ciRS-7 was largely depleted in the enriched circRNA pool from mouse while it was covered by >400 long BSJ-spanning reads in the human sample. This is likely explained by the presence of several internal poly(A) stretches in ciRS-7 that can bind the oligo-dT beads used for poly(A) RNA depletion. Indeed we found that circRNA with stretches of 7 or more “A” residues was partially depleted with 33% and 56% in the top 1000 detected circRNAs (in the long-reads versus the short-reads datasets) in human and mouse brains, respectively (Supplementary Data 17–18). However, we noticed that ciRS-7 levels also vary between human and mouse brain in our short-read sequencing data (511 BSJ reads in human vs 24 BSJ reads in mouse). Mouse ciRS-7 also appeared more sensitive to RNase R, possibly due to its larger size (2927 nts vs 1485 nts in human), as previously observed for other circRNAs^32^.

Our circRNA enrichment protocol furthermore revealed that the *βIII-tubulin* mRNA is 10-fold more resistant to RNase R treatment than the *Eef1a1* mRNA, presumably due to differences in secondary structure. Further investigation is needed to see how RNase R treatment affects different linear and circular RNAs on a global scale. Until then, we need to stress that circRNA purification protocols can create significant biases and that care must be taken to only compare RNA profiles created with the same purification strategy.

To get a deeper view of the alternative splicing landscape of selected, multi-exonic circRNA, we developed circPanel-LRS. This approach does not require circRNA enrichment, making it valuable for low-input samples. The need for gene-specific primers makes this technique ideal for delineating the complexity of circRNAs identified in global screens, from circRNA databases or computational predictions for a region of interest.

The circPanel-LRS method involves an RT enzyme with strand displacement activity and it can therefore generate long-reads that derive from multiple ‘rounds’ of RT activity within the same circRNA (Supplementary Fig. 4). The formation of circRNA concatemers during reverse transcription is a matter of active discussion in the field since it can lead to the overestimation of circRNA levels when using standard quantification methods such as qPCR or short-read RNA sequencing. In contrast, the presence of RT-induced concatemers is readily detected by long-read sequencing.

Our data also enabled a full-length sequence analysis of intron-containing circRNAs known as exon–intron circRNAs (EIciRNAs) in mouse and human brain, some of which were species specific (Supplementary Data 5 and 6 and Fig. 3a, b). These RNAs have been reported to be localized in the nucleus and in some instances increase the expression level of their host gene^40^. This contrasts intron retention in mRNA, which generally represents inefficient splicing and triggers nuclear retention and degradation. However, some retained introns are known to regulate gene expression by nuclear retention^41^ and produce new isoforms with specific functions^42^. Our data do not distinguish nuclear from cytoplasmic circRNA and it remains unclear to what extent intron retention could regulate cellular compartmentalization of circRNA and whether they are associated with regulatory functions.

A large number of alternative splicing events were detected using circNick-LRS and, in particular, when using the higher sequence coverage in circPanel-LRS. Another observation from our long-read sequencing data is the high frequency of unannotated exons found in circRNAs. So why do we observe so many unique and cryptic circRNA exons not normally seen in linear spliced mRNA? One explanation may be that the circRNA-specific exons are also initially spliced into linear mRNA but that the resulting transcripts are more likely to be prone to degradation in the cytoplasm by nonsense-mediated decay (NMD). The NMD pathway is known to target mRNAs carrying a premature termination codon (PTC) in a translation-dependent manner^43^ and, particularly in the brain, NMD is linked to development and neurodegenerative disorders^44,45^. The insertion of non-coding exons between coding exons in linear mRNA is likely to cause NMD, either by shifting the reading frame or by introducing a stop codon. In contrast, since circRNAs are generally not translated^46^, they will not be subjected to NMD and can therefore tolerate the inclusion of novel exons. In support of this theory, more than 76% and 82% of novel circRNA-specific exons in mouse and human brain, respectively, are either predicted to introduce frame-shifting or contain at least one in-frame stop codon (Fig. 6a–d).

Another possible explanation for the increased occurrence of novel exons in circRNAs could be that the novel exons are only spliced into the circular form of the RNA. The circRNA-specific exons may bind splicing factors or introduce RNA structures that selectively stimulate back splicing events rather than forward splicing. Such a system could function as a proofreading system to correct mRNA splicing by circularizing aberrantly spliced exons. Further investigation is needed to determine whether the alternative circRNA exon structure influences the sub-cellular localization and hence also its potential function. It is also unclear whether circRNA splice-variants are co-expressed in the same cells or originate from different cells in the brain.

Interestingly, a large number of microexons were observed in both human and mouse data (Fig. 6e–h). Microexons generally contain 3–29 nts and are important regulators of the transcriptome, especially in neurogenesis where splicing factors such as nSR100/SRRM4, RBFOX, and PTBP1 regulate the inclusion of brain-specific microexons^38,47,48^. Deeper analysis of our data revealed that some microexons are mainly or exclusively found in circRNAs. In particular, we noticed that several annotated microexons previously linked to autism^38,39^ are preferably found in the circRNA generated from the host gene. Previous studies on microexon annotation relied on short-read RNA sequencing data and therefore misannotated them to linear mRNA transcripts. The observation that several microexons linked to neuronal development and autism are mainly expressed in circRNAs questions their mechanism of action since circRNAs are unlikely to undergo translation^46^. We speculate that the microexons in circRNAs could function as transcription regulators or binding sites for miRNAs and/or proteins^49,50^.

While this study was in revision, two other papers reported the use of nanopore long-read sequencing of circRNAs^51,52^. The isoCirc approach published by Xin et al. initiates cDNA synthesis using random hexamers followed by rolling circle amplification to generate concatemers. Notably, this protocol appears to generate shorter average lengths for the BSJ-containing consensus sequences (300–460 nts) compared to circNick-LRS (average of 795 nts for full-length detected circRNAs in human brain; Supplementary Fig. 7). As an example, circFAT3 (circBase ID: hsa_circ_0000348), a 3,300 nts circRNA highly expressed in the brain, was not detected by isoCirc. Additionally, circPanel-LRS generates full-length cDNA from targeted circRNAs of any size and also makes concatemeric reads from the same circRNA template for better validation. The main difference between isoCirc and circPanel-LRS, in terms of cDNA synthesis and library enrichment, is that circPanel-LRS uses circRNA-specific primers while isoCirc relies on random hexamers. Xin et al. report a relatively high number of new circRNAs isoforms but this may reflect that they only compare their data to circBase and the cancer-specific MiOncoCirc circRNA database whereas we use the more comprehensive databases circAtlas, CIRCpedia, and circBase^33–35^. Furthermore, we note that Xin et al. seem to have misinterpreted our original work on bioRxiv^26^ neglecting that calling a circRNA requires the presence of BSJ-containing reads. Additionally, only circRNA-specific splicing events that meet a defined read coverage of 5, 10, or 20 reads, depending on the type of analysis, were included in our analysis. The relative high number of reads per circRNA allowed us to investigate exon composition, novel exons, alternative exon usage, intron retention, and microexons in thousands of circRNAs.

During the revision of our manuscript, Zhang and colleagues also reported a method for nanopore sequencing of circRNAs called CIRI-long^52^. Here, cDNA synthesis is initiated using random hexamers that amplifies the circRNA signal by forming concatemers of the same circRNA at the RT step and subjecting this to direct cDNA sequencing after library size selection. The average length distribution of the detected full-length circRNAs generated by CIRI-long is <500 nts, which is also shorter than 795 nts, the average size of full-length detected circRNAs generated by circNick-LRS approach in human brain sample. Considering the length distribution of the raw reads and the copy number of the concatemeric cDNA sequences, Zhang et al., have mainly covered small circRNAs. Hence, it appears that the shorter reads obtained with both isoCirc and CIRI-long may be a consequence of premature termination caused by annealing random hexamers when an RT enzyme with weak strand displacement and having RNase H activity is used. In contrast, circNick-LRS relies on circRNA fragmentation, followed by polyadenylation before cDNA synthesis. The fragmentation step in the circNick-LRS protocol was optimized to primarily cut once, even in larger circRNAs, to allow full-length circRNA reads. Since larger circRNAs are more likely to be nicked this may create a bias of our method towards longer circRNAs. In addition, we apply a size cut-off in the cDNA amplification step where circRNAs <200 nts are depleted. These technical biases are avoided in circPanel-LRS where the primer design ensures full-length sequencing of circRNAs without prior circRNA enrichment, something that will be beneficial for labs working with a group of preselected circRNAs.

Importantly, we sequenced both circRNA and linear poly(A) RNA from the same RNA samples, enabling a direct comparison of exon usage in the two groups of RNAs and allowing us to identify and quantify specific splicing events in both types of RNA.

In conclusion, our circRNA enrichment strategy combined with nanopore mediated long-read sequencing allows a detailed characterization of full-length circRNAs in all size ranges. The most striking observation from our study was the large number of circRNA-specific splicing events found in human and mouse brain tissue, which illustrates the importance of long-read sequencing. It remains to be seen whether the unusual splicing pattern of circRNAs merely reflects the absence of NMD or whether alternatively spliced circRNAs mediate new functions independently of the linear host transcript. The latter option is underscored by the notion that several microexons linked to neurodevelopmental disorders are preferentially found in circRNAs, calling for a reevaluation of their mechanism of action.

## Methods

In order to cover the circular RNAs derived from both X and Y chromosomes, total RNA was obtained from male gender for both human and mouse samples. Total human brain RNA was derived from post mortem cortex and provided by Agilent (Agilent Technologies, cat: 540143). For the preparation of mouse brain RNA, a 2 months old male mouse (strain 129S2/SV) was killed and the entire brain was harvested and grained in Trizol (Thermo Fisher Scientific). Total RNA was obtained according to the manufacture’s protocol. Animals were treated according to the regulation of “The Animal Experiments Inspectorate”, the legal authority under the “Ministry of Environment and Food of Denmark” (https://www.foedevarestyrelsen.dk/english/Animal/AnimalWelfare/The-Animal-Experiments-Inspectorate/Pages/default.aspx). All mice had free access to food and water supplied ad libitum and were kept under 14-h light/10-h dark cycle and standard conditions of ambient temperature (22 °C ± 1 °C) and humidity (50% ± 10%).

DNA LoBind tubes (Eppendorf) were used for all steps during the RNA preparation and ONT cDNA-PCR library construction. RNA and dsDNA concentrations were determined using a Qubit 4.0 fluorometer together with the Qubit HS dsDNA and Qubit HS RNA kits (Thermo Fisher Scientific). RNA quality was assessed after each step of circRNA preparation and polyadenylation using Agilent 2100 bioanalyzer (Agilent Technologies). DNase I treatment was utilized to remove DNA from both the human and mouse total RNA preparations. The quality of total RNA samples was confirmed by agarose gel analysis and Agilent 2100 bioanalyzer.

### Total RNA short-read sequencing

Human and mouse brain samples were used for short-read total RNA sequencing. The rRNA was depleted and sequencing was performed using DNA nanoball sequencing technology and BGISEQ-500 sequencing platform and 30 million paired-ends (PE100) reads were generated per sample.

### Preparation of linearized polyadenylated circRNA for nanopore sequencing

Generally, RNA Clean and Concentrator^TM^ -5 (R1016, Zymo Research) was applied after each step in the RNA preparation procedure to clean up and concentrate the RNA, using the adjusted RNA Binding Buffer to select only RNA transcripts longer than 200 nts. RiboLock RNase Inhibitor (Thermo Fisher Scientific, EO0381) was added to the eluted RNA to prevent RNA degradation after each step. First, the Ribo-Zero rRNA Removal Kit (Human/Mouse/Rat, Illumina) was employed to deplete ribosomal RNA from 20 µg of total human or mouse brain RNA according to the manufacture’s instruction. Then, the rRNA-depleted sample was treated with RNase R (Epicentre, RNR07250) to digest all linear RNAs which are not resistant to degradation by RNase R. In order to further enrich the ratio of circRNAs to linear RNA, the remaining linear RNAs were polyadenylated utilizing the Poly(A) Polymerase (New England Biolabs, M0276) according to the manufacturer’s instruction and subsequently removed with NEBNext Poly(A) mRNA Magnetic Isolation Module (New England Biolabs, E7490S) following the kit’s manual. Supernatant obtained from the first step of the poly(A) purification contained the Enriched CircRNA Pool (ECP) and was used in the next step.

Next, a NEBNext Magnesium RNA Fragmentation Module (New England Biolabs, E6150) was used to linearize the circRNAs obtained from ECP and in preparation for sequencing. In order to fragment both small and large circRNAs, and prevent over-degradation of large circRNAs, the ECP sample was divided into three aliquots and subjected to 80 °C incubation for 30 s, 1 or 2 min, respectively, before pooling them again. After fragmentation, the 3´phosphate group was removed from linearized circRNAs, and phosphate groups added to the 5´ends using T4 Polynucleotide Kinase (New England Biolabs, M0201S) by first incubating for 30 min without ATP and then 30 min with ATP to remove and add the phosphate group, respectively. Finally, the linearized RNAs were polyadenylated as described above and used as input for the cDNA-PCR sequencing procedure (Fig. 1a, b). We named this strategy circRNA Nicking and Long-Read Sequencing (circNick-LRS).

### Evaluation of circRNA abundance during RNA preparation

For cDNA preparation and to evaluate the efficiency of circRNA enrichment in ECP and for microexon validation by RT-PCR, we used the Superscript VILO cDNA Synthesis Kit (Thermo Fisher Scientific) according to the manufacturer’s protocol. The LightCycler 480 SYBR Green I Master Kit was used for the qPCR reactions. *βIII-tubulin* was chosen as housekeeping gene, 18S ribosomal RNA as a marker for rRNAs and circHipk3 to represent the circRNA. To do RT-PCR for microexon validation, LongAmp Hot Start Taq 2X Master Mix (New England Biolabs, M0533L) was used following the manufacturer’s instruction. Sequence of the primers provided in Supplementary Table 1.

### CircNick-LRS cDNA-PCR sequencing

The manual of cDNA-PCR Sequencing Kit (SQK-PCS108, version PCS_9035_v108_revF_26Jun2017), provided by Oxford Nanopore was applied with a few modifications as described below.

### Reverse transcription and strand-switching

Briefly, 9 µl, equal to 9 ng of polyadenylated ECP RNA, 1 µl VNP primer (ONT), and 1 µl 10 mM dNTPs were mixed and incubated at 65 °C for 5 min and snap-cooled on a pre-chilled freezer block. Then, a mix of 4 µl Superscript IV buffer (Thermo Fisher Scientific), 1 µl RNaseOUT, 1 µl 100 mM DTT, and 2 µl Strand-Switching Primer (SSP, ONT) was added to the cold sample, followed by incubation at 42 °C for 2 min. Finally, 1 µl of 200 U/µl Superscript IV Reverse Transcriptase (Thermo Fisher Scientific) was added and the following protocol completed using a thermocycler: One cycle at 50 °C for 10 min (reverse transcription), one cycle at 42 °C for 10 min (strand-switching), one cycle at 80 °C for 10 min (heat-inactivation) and cool down to 4 °C.

### PCR amplification of cDNA

A set of 4 × 50 µl reactions were prepared, each containing 25 µl LongAmp Taq 2X Master Mix (New England Biolabs, M0287S), 3 µl cDNA PRM primer (cPRM, ONT), 17 µl nuclease-free water, and 5 µl reverse-transcribed RNA sample. The cycle steps of the PCR were: (1) 95 °C for 30 sec, (2) 95 °C for 15 sec, (3) 62 °C for 15 sec, (4) 65 °C for 6 min, repeat steps 2–4 for 18X, (5) 65 °C for 6 min and (6) cool down to 4 °C. Each PCR reaction was treated with 1 µl of Exonuclease I (New England Biolabs, M0293S) at 37 °C for 15 min followed by 80 °C for 15 min to inactivate the Exonuclease I enzyme.

### Agarose gel purification and size selection of the PCR products

A set of 4 × 40 µl of PCR reaction was mixed with 8 µl 6X DNA load dye (Thermo Fisher Scientific) and run on a 2% TBE agarose gel (UltraPure, Thermo Fisher Scientific) for 2 h at 80 V. Using the 100 bp and 1 kb DNA Quick-Load ladders (New England Biolabs, N0467 and N0468) as a reference, the PCR products in the range from 350 bp to 10 kb were cut out and extracted from the gel (GeneJET Gel Extraction Kit K0691, Thermo Fisher Scientific).

### Re-amplification of the gel-extracted library

The 350 bp–10 kb products were subjected to PCR amplification and Exonuclease I digestion as described above, except that 2 µl cPRM and 6 cycles of steps 2–4 was applied. In order to confirm the quality of amplified DNA, 5 µl of the PCR product was analyzed in a 2% TBE agarose gel. The remaining ~200 µl PCR material was mixed with 160 µl AMPure XP beads (Beckman Coulter) followed by rotation for 5 min at room temperature. The beads were subsequently washed twice using 500 µl fresh 70% ethanol, and DNA material eluted from the beads using 21 µl of Rapid Annealing Buffer (RAB, ONT) during 10 min rotation at room temperature. To add the adapter, 400 fmol of the eluted DNA was adjusted to 23 µl using RAB buffer and used for adapter ligation by addition of 2 µl cDNA adapter mix (cAMX, ONT) and incubating for 5 min at room temperature with rotation. The mixture was purified using 20 µl of AMPure XP beads, incubated for 5 min at room temperature with rotation, followed by washing the beads twice with 140 µl of Adapter Bead Binding Buffer (ABB, ONT). Elution was done with 13 µl of elution buffer (ELB, ONT) for 10 min at room temperature with rotation. Then 12 µl of the ELB eluate was mixed with 35 µl of Running Buffer with Fuel mix (RBF, ONT), 25.5 µl of Library Loading Beads (LLB, ONT), and 2.5 µl of nuclease-free water, and the sample sequenced for 48 h using the MinION Mk1B with a FLO-MIN106 R9 Flow Cell.

### CircRNA Panel Long-Read Sequencing (circPanel-LRS)

A more efficient and convenient approach was developed for long-read and multiplex sequencing and splicing events analysis of an already selected panel of circRNAs (circPanel-LRS). The circPanel-LRS protocol was adapted to target and sequence a panel of selected circRNAs directly using total RNA without need of any prior circRNA enrichment and with the possibility of multiplexing different samples combined and sequenced in one round.

A panel of 10 circRNAs was selected based on sequencing data obtained from the circNick-LRS long-read approach and also short-reads data from the human brain sample. The circRNAs were selected from different sizes in length and from low to high expression (Supplementary Data 3). As shown in Fig. 1c, 25 nts length primers were designed for reverse transcription to anneal either 30 nts down of the BSJ or 5′ end of the mostly shared exon, covering as many as possible different circRNA isoforms obtained from the same host linear. Primers were designed at least with 40% GC and Tm of more than 60 °C in the LongAmp Hot Start Taq 2X Master Mix buffer (New England Biolabs, M0533L). These primers were extended at the 5′ end to be targeted by PCR amplification and samples barcoding primers and to be adjusted to the ONT cDNA-PCR sequencing protocol with (SQK-PCB109) or without (SQK-PCS109) sample barcoding (Supplementary Data 3).

For cDNA synthesis, Maxima H Minus Reverse Transcriptase (Thermo Fisher Scientific, EP0752) was used and a total of 10 preselected circRNAs loci were targeted. Briefly, 1 μg of DNase I treated total RNA, 1 μl of a 10 pmol primer mix (pool of 10 primers containing 1 pmol of each primer), and 1 μl 10 mM dNTPs (2.5 mM each) pooled and up to 14 μl nuclease-free water was added. RNA-Primer mix was incubated at 70 °C for 3 min and snap cooled down on ice. Later, 4 μl 5x RT Buffer for Maxima H Minus Reverse Transcriptase (Thermo Fisher Scientific) and 1 μl RiboLock RNase inhibitor (40 U/μl) were added and incubated at 42 °C for 2 min. Gently, 100 U Maxima H Minus RT enzyme was added and incubated for 30 min at 50 °C, followed by 5 min at 85 °C to inactivate the RT enzyme and kept at 4 °C. Afterward, 1 μl RNase H (Thermo Fisher Scientific, EN0201) and 1 μl RNase Cocktail Enzyme Mix (Thermo Fisher Scientific, AM2286) were added and incubated at 37 °C for 20 min and followed by 20 min at 75 °C, then kept at 4 °C. The second strand of the cDNA was made using back-to-back panel circRNA-specific divergent primers. The final 22 μl cDNA of each sample from previous step, was divided into two 0.2 ml PCR tubes and the following materials were added to each tube separately including 25 μl LongAmp Hot Start Taq 2X Master Mix (New England Biolabs, M0533L), 1 μl of the 10 pmol second strand cDNA synthesis primer mix (1 pmol of each primer) and 13 μl nuclease-free water added up to the final of 50 μl. In order to synthesize the second strand of the cDNA and to enrich the targeted circRNA isoforms, the PCR reactions prepared in the previous step were incubated for 1 cycle of 2 min at 95 °C, 1 min at 50 °C, and 10 min at 65 °C, followed by 2 cycles of (1) 95 °C for 15 sec, (2) 55 °C for 15 sec, (3) 65 °C for 5 min, and followed by 6 more cycles of (1) 95 °C for 15 sec, (2) 62 °C for 15 sec, (3) 65 °C for 5 min, and one cycle of 5 min incubation at 65 °C and cool down to 4 °C.

Each PCR reaction was treated with 1 µl of Exonuclease I (New England Biolabs, M0293S) and incubated at 37 °C for 15 min to digest any unincorporated primers and incubated at 80 °C for 15 min. The PCR products were cleaned up using SPRIselect beads and the library was subjected to 14 cycles of PCR using barcoded and ONT-adjusted primers (cPRM primers) from the SQK-PCB109 kit. Briefly, the total of 100 µl cDNA-PCR product per sample pooled and 65 μl well-mixed SPRISelect beads was added (0.65X) to each tube, mixed and rotated for 10 min at room temperature. Later, the beads were pelleted and washed twice using 500 µl freshly prepared 80% Ethanol. The pellet was dried for 2.5 min, resuspended in 25 μl nuclease-free water by pipetting and incubated for 5 min at 37 °C followed by 5 min rotation at room temperature. The beads were pelleted for 5 min on a magnetic rack and the supernatant containing the purified library was harvested. Later, the second round of the PCR was applied using barcoded cPRM primers from the ONT SQK-PCB109 kit. For each sample 4 × 0.2 ml PCR tubes were prepared each containing 2 ng cDNA library from the previous PCR, 25 µl LongAmp Hot Start Taq 2X Master Mix (New England Biolabs, M0533L), 1.5 µl cDNA PRM primer (cPRM, ONT) provided for SQK-PCB109 kit, and nuclease-free water was added up to 50 µl. The PCR reactions were incubated for 1 cycle of 2 min at 95 °C, followed by 14 cycles of (1) 95 °C for 15 sec, (2) 62 °C for 15 sec, (3) 65 °C for 5 min, and one cycle of 5 min incubation at 65 °C and kept at 4 °C. Exonuclease I treatment was applied and the PCR products were cleaned up using SPRISelect beads as explained for the first PCR round, except that the library was eluted in 15 µl elution buffer (EB, provided by ONT SQK-PCB109 kit). Quantity, quality, and purity of the libraries were assessed using Qubit 4, Agilent 2100 bioanalyzer, and Nanodrop respectively.

A mix of an equimolar ratio of libraries were pooled and multiplexed up to a total of 100 fmol and used for ONT sequencing adapter ligation and ONT MinION sequencing following the manufacturer’s instruction for the SQK-PCB109 kit.

### Data analysis

Nanopore data was basecalled using Guppy (v 3.4.5), which included barcode trimming. Fastq files produced by Guppy were filtered using NanoFilt (v 2.6.0) allowing only reads with average base quality of 7 or more and a read length of 250 bp or more. The filtered data was mapped to the human genome (hg19) or mouse genome (mm10) using a parallelized version of Blat software package^53,54^. Blat is able to map sequences across linear splice events but when encountering a BSJ splicing event, it splits into two segments that individually can be mapped to the genome. The observation of two segments that map upstream of a splice donor and downstream of a splice acceptor, respectively, signifies a BSJ and can hence be interpreted as a circRNA. A Blat score is calculated as the number of matching nucleotides subtracting mismatches and gap penalties. To qualify as a circRNA, the Blat score has to reach 30 on both sides of the BSJ. A blat score of 30 is a stringent requirement, so only a single read fulfilling this criterium is required to define a circRNA in this study. A putative circRNA is reported if the following criteria are met: the two hits from the same read must be (1) on the same genomic strand, (2) within 1 Mb, (3) must not overlap by 50 or more bp, and (4) must be oriented in reverse order relative to the read sequence.

Reads that fulfill the above-mentioned criteria are annotated with the number of bp overlapping a refSeq gene, exon of refSeq gene, Expressed sequence tags (ESTs) from the UCSC genome browser and introns of refSeq genes. This information is stored for each single read and is used later to assess the intron usage level in each detected circRNA. For visualization and further analysis, psl files were converted to bed12 format using a custom script and to bam format using samtools.

Using custom scripts, hits originating from the same read were combined, only allowing reads with exactly two hits marking the genomic ends of circRNAs. The previously added gene/exon/EST/intron bp annotation data is summed when read hits are combined. Reads mapping to mitochondria and rRNA genes are excluded from this analysis. Since the ends of these putative circRNA reads often are heterogeneous due to the data quality of the Nanopore 1D reads, end-processing of the reads is performed by searching for the closest refSeq exon using bedtools. If both ends of a putative circRNA are within 30 bp of an annotated refSeq exon, the end-sequences of the putative exon are corrected accordingly. This effectively removes the end heterogeneity of the Nanopore data, when reads originate from RefSeq annotated genes. Putative circRNAs that do not satisfy this requirement are matched against circRNAs annotated on circBase, circAtlas, and CIRCpedia. If a read has 95% overlap in its genome mapping position to an annotated circRNA, the end-sequences of the putative circRNA are corrected to fit the annotated circRNA ends. All reads satisfying the two adjustment criteria are accepted as circRNA candidates.

CircRNA candidate reads are collapsed to show only one unique genomic region for each position found to produce a circRNA. These unique circRNA regions are annotated with overlapping gene (host gene), circBase ID, circAtlas ID, and CIRCpedia ID where available, the count of candidate reads that map to the unique region, as well as the mean value of the previous gene/exon/EST/intron bp annotations for the mapping reads. Finally, the minimum, maximum, and mean number of bp the mapping reads were adjusted to fit the mapping circRNA genomic region is added.

Fastq quality scores are defined as *Q* = −10 × log(err), where err is the per base error-rate. The error-rate can be calculated as: err = 10^(−*Q*/10). Example for quality score of 11.95: err = 10^(−11.95/10) = 0.064 = 6,4%. NanoPlot (v 1.29.0) was used to visualize read length and quality.

Custom perl and bash scripts can be found on github (https://github.com/omiics-dk/long_read_circRNA).

### Conservation of circRNAs

Conservation of circRNAs between mouse and human brain samples was examined using the UCSC liftOver tool as described in Veno et al.^2^. Twenty base pairs from each end of circRNAs were lifted from mm10 to hg19 or from hg19 to mm10, recombined and compared to circRNAs sequenced from the relevant species. This approach allows conversion of circRNA BSJ genomic coordinates between mouse and human genomes without requirements for internal splicing patterns of the circRNAs. CircRNAs are conserved when conversion between mouse and human genomes have exact matches to detected circRNAs in both datasets.

### Detection of intron usage in circRNAs

In order to detect potential intron retention in circRNAs, we detect the percentage of intron mapping bases from BSJ spanning reads for each annotated intron within circRNA genomic regions. This data was collected for each read individually, showing how many bp each individual BSJ spanning read maps to Gencode annotated exons and introns. This allows us to calculate the intron coverage for each circRNA, defined as the mean number of intron mapping bp in the BSJ spanning reads that define the circRNA divided by the mean number of gene mapping bp in the BSJ spanning reads.

Introns within circRNA genomic regions that are covered by 20 or more BSJ reads, have read coverage across 90% or more of the intron length and no detection of previously unannotated (novel) exons are called as retained introns.

### Novel exons

Exons present in circRNAs but not annotated in either Gencode or RefSeq were detected by scanning mapped Nanopore reads for flanking AG-GT splicing signatures in the reference genome. Read segments mapping in discrete blocks, with allowance of up to 10 bp internal deletion, were checked for flanking base pairs. Segments that are flanked by AG on the 5’ side and GT on the 3’ side, consistent with RNA splicing, are marked as likely exons. If two or more reads corroborate the exon definition, it is considered a bona fide exon. No end processing of the Nanopore reads is done before detecting flanking sequences. Detected circRNA exons that do not match an annotated exon by at least 95% similarity on genome coordinates are considered novel exons.

Note that only circRNAs with two or more exons contribute to this analysis, since BSJ spanning reads are required to completely cover an exon in a single genomic mapping location. Single-exon circRNAs are combined from two genomic mapping locations of individual BSJ spanning reads, and do not count in this analysis.

Novel exons that do not overlap any annotated exon are classified as “unique exons”, whereas exons that partially overlap an already annotated exon are classified as “cryptic exons”, since these exons presumably arise from cryptic splice sites not normally used in the linear mRNA host (Fig. 5a).

Novel exons are investigated for their ability to introduce stop codons. In order to do this, the codons for each of the three possible phases were prepared for all novel exons and the presence of stop codons in each of the three phases was detected. By use of a custom script Gencode gene annotations were processed to get the phase used by each annotated exon. The phase used by the immediate upstream exon dictates the phase used by a novel exon. If the phase in use by a novel exon contains a stop codon, insertion of this exon in a protein coding mRNA would disrupt transcript function, but insertion in a non-protein-coding circRNA such a stop codon would have no consequence.

### Alternative exon usage

The usage of exons within the mapping range of each individual BSJ spanning read is detected and used to build alternative exon usage tables for each circRNA. For each circRNA, we quantify the number of BSJ reads mapping to genomic coordinates, including specific exons and how many of these BSJ reads have sequence mapping to the exon. Exon usage level is then defined as the number of BSJ reads using the exon divided by the number of BSJ reads mapping across the exon. Only exons used in 10 or more reads for circNick-LRS or 50 or more for circPanel-LRS are included in the analysis. Exons that are constitutively used in circRNAs have an exon usage level of 1, whereas alternatively used exons have an exon usage level between 0 and 1.

### FLAIR analysis of ONT cDNA-PCR sequencing data

Data was basecalled using Guppy (v 3.4.5). Basecalled fastq files were analyzed using FLAIR v 1.4 (Full-Length Alternative Isoform analysis of RNA)^30^, performing genome alignment and end correction of the ONT cDNA-PCR reads. The corrected reads were collapsed to generate complete lists of isoforms detected.

### Short RNA sequencing data analysis

Adapter sequences and low quality reads were trimmed away using Trim Galore (v 0.4.1). Short RNA sequencing data was quality controlled using FastQC (v 0.11.5). Detection of circRNAs was primarily done using CIRI2 (v 2.0.6) and secondarily by find_circ (v 1), which was used to corroborate circRNA detection by CIRI2. Positive detection of circRNAs by two different algorithms is know to reduce false circRNA detection.

### Reporting summary

Further information on research design is available in the Nature Research Reporting Summary linked to this article.

## Supplementary information

Supplementary Information

Supplementary Data 1

Supplementary Data 2

Supplementary Data 3

Supplementary Data 4

Supplementary Data 5

Supplementary Data 6

Supplementary Data 7

Supplementary Data 8

Supplementary Data 9

Supplementary Data 10

Supplementary Data 11

Supplementary Data 12

Supplementary Data 13

Supplementary Data 14

Supplementary Data 15

Supplementary Data 16

Supplementary Data 17

Supplementary Data 18

Reporting Summary

## Data Availability

The data supporting the findings of this study are available from the corresponding authors upon reasonable request. All the sequencing data generated in this study have been deposited to the Gene Expression Omnibus (GEO) repository database with the accession number GSE127059. The following circRNA databases were used: circBase (http://www.circbase.org/), CIRCpedia (https://www.picb.ac.cn/rnomics/circpedia/), and CircAtlas (http://circatlas.biols.ac.cn/)^33–35^. Source data file is provided.

## Code availability

Custom scripts used in this manuscript and a minimal test dataset can be found on github: https://github.com/omiics-dk/long_read_circRNA.
