# Supplementary material for: Nanopore sequencing of brain-derived full-length circRNAs reveals circRNA-specific exon usage, intron retention and microexons
Source: Nat Commun. 2021 Aug 10;12:4825. doi: 10.1038/s41467-021-24975-z (PMC8355340; doi:10.1038/s41467-021-24975-z)
Supplement: Supplementary file 1 — Supplementary Information [file 41467_2021_24975_MOESM1_ESM.pdf]

Supplementary Information

Nanopore sequencing of brain-derived full-length circRNAs reveals circRNA-specific exon usage, intron retention and microexons

Karim Rahimi[*1,2], Morten T. Venø[1,2,3], Daniel M. Dupont[2], Jørgen Kjems[*1,2]

[1]Department of Molecular Biology and Genetics (MBG), Aarhus University, DK-8000, Aarhus, Denmark.

[2]Interdisciplinary Nanoscience Center (iNANO), Aarhus University, DK-8000, Aarhus, Denmark.

[3]Omiics ApS, DK-8200, Aarhus, Denmark

*Correspondence:jk@mbg.au.dk; karim@mbg.au.dk

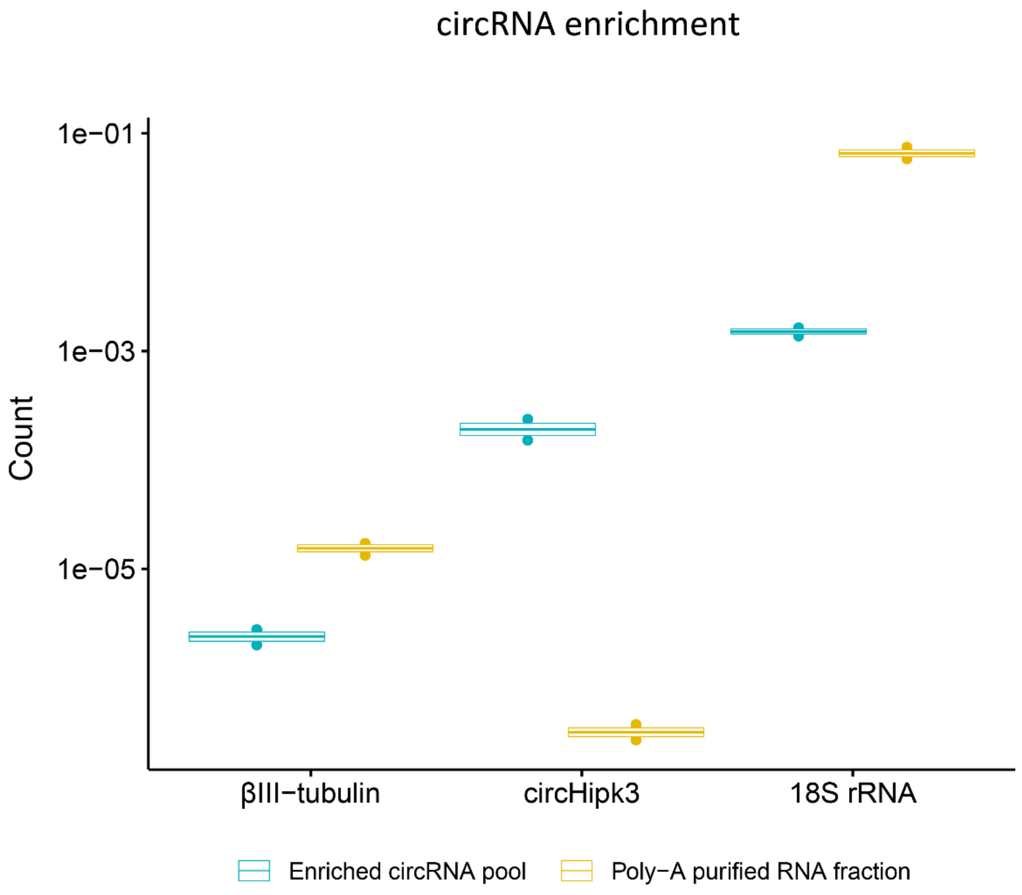

Supplementary Figure 1: CircRNA enrichment.

After RiboZero and RNase R treatment, the remaining linear RNA was polyadenylated and depleted by oligo-dT beads. The enriched circRNA pool (cyan) represents the supernatant, and the poly-A purified RNA fraction (mustard) represents the eluate from the oligo-dT beads which represents mRNA and all other polyadenylated linear RNA. The enrichment efficiency was determined by qPCR for the relative amount of neuron-specific housekeeping gene βIII-tubulin, circRNA circHipk3 and 18S rRNA. The circRNA level was significantly enriched in the supernatant obtained from the pelleted oligo-dT beads compared to the poly(A) purified. CircHipk3 has been enriched more than 100x compared to βIII-tubulin, but still 10x less than 18S rRNA in the final circRNA pool.

The mean values are shown as bold horizontal lines and hinges represent the standard error of the mean. Mean represent the average of 2^-Ct obtained from three technical replicates. Individual data points are shown. The y-scale is logarithmic. Colored by preparation (“circRNA enriched pool” or “Poly−A purified RNA fraction”).

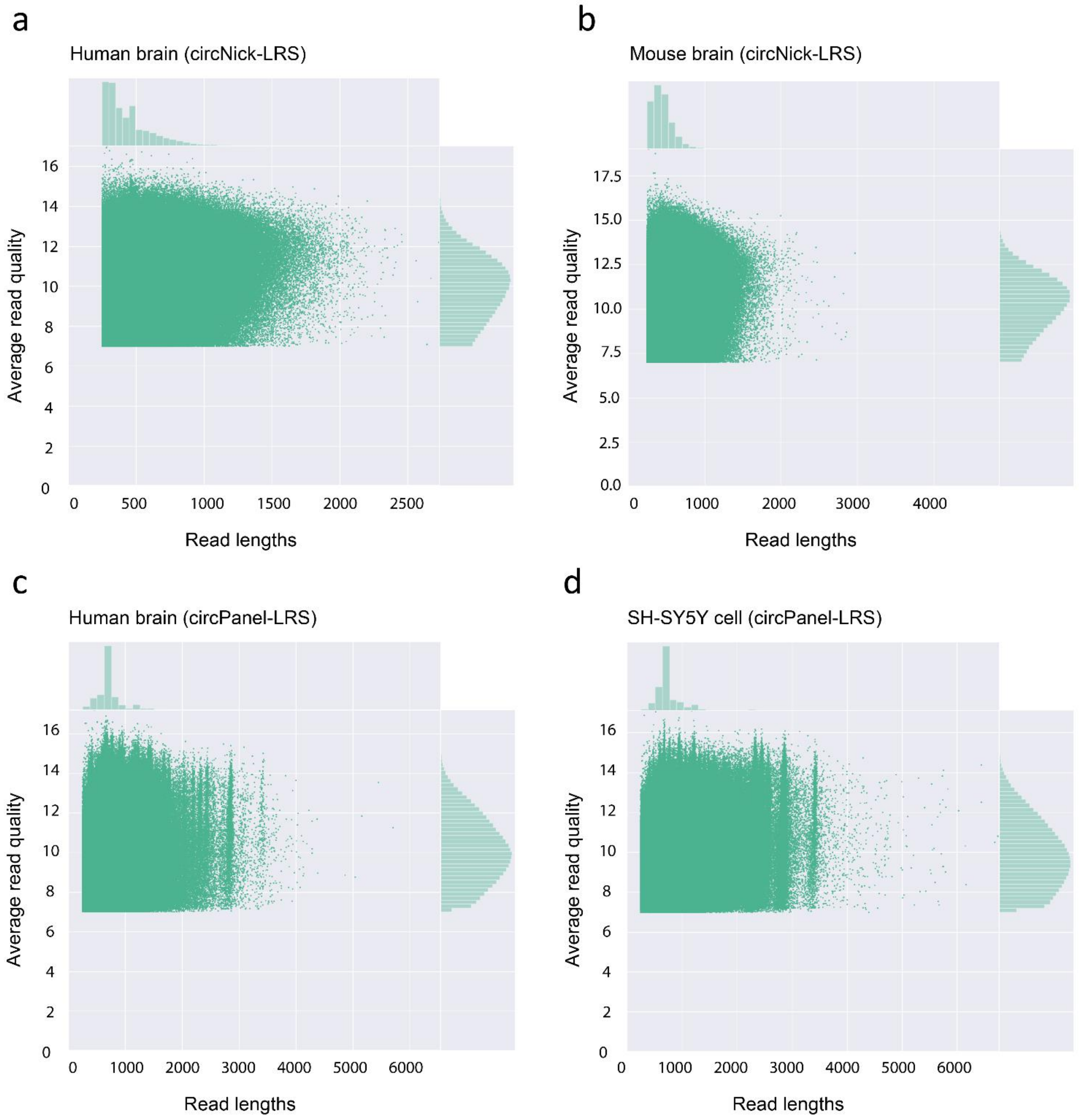

Supplementary Figure 2: Nanopore 1D circRNA sequencing metrics.

a-d) Scatter plot of read length versus average read quality for all four circRNA sequencing datasets obtained by Nanopore MinION sequencing.

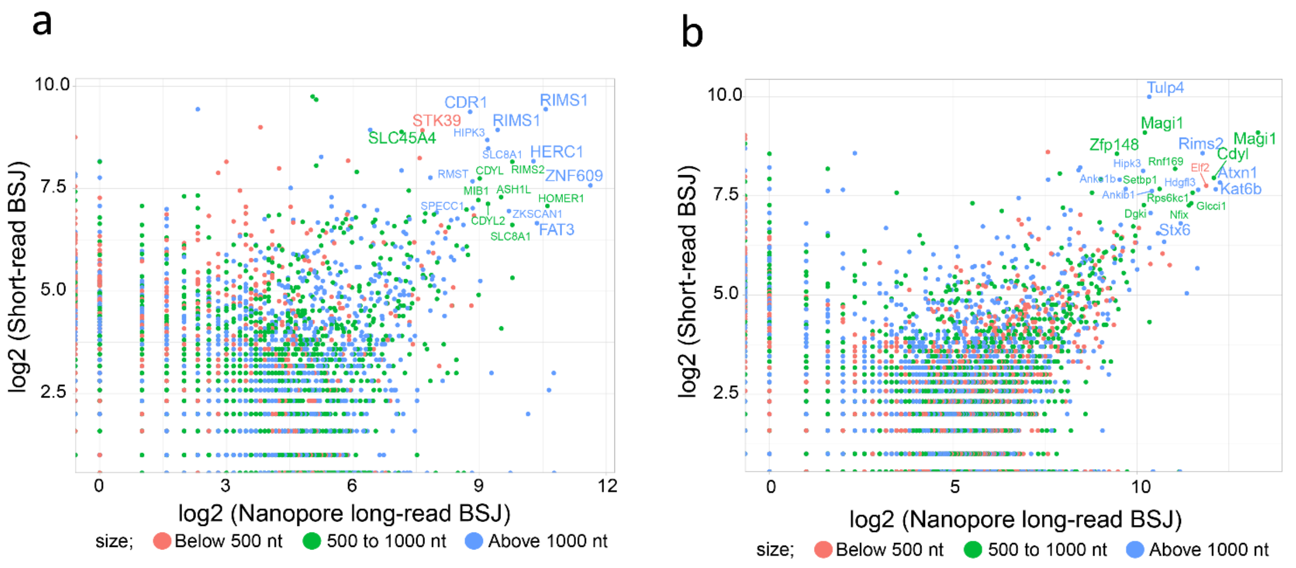

Supplementary Figure 3: CircRNA detection in human and mouse brain samples using short- and long-read sequencing technologies.

CircRNAs detected in the human **(a)** and mouse **(b)** brain samples. Short read data (Y-axis) vs long-read data (X-axis) indicates that both technologies detect BSJ-containing reads of highly expressed circRNA but more selective for lower expressed circRNAs.

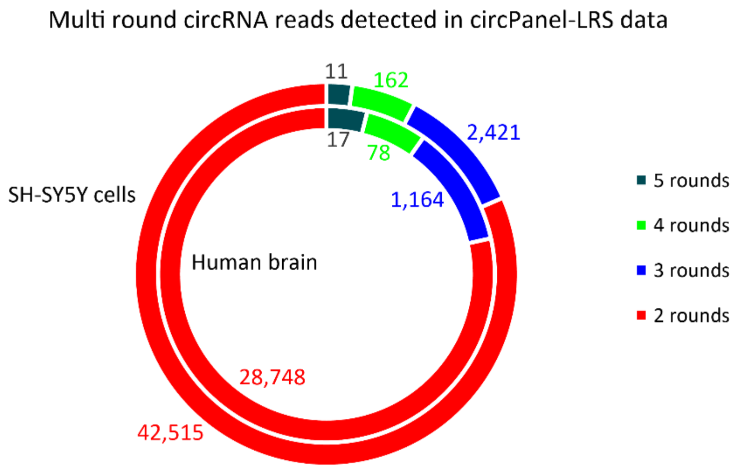

Supplementary Figure 4: Multi round circRNA reads obtained from circPanel-LRS approach.

Multi-round circRNA reads detected in the circPanel long-read sequencing data, probably due to strand displacement activity of the reverse transcriptase enzyme. The cDNA covered the circRNA sequence more than one round, and up to 5 rounds were detected.

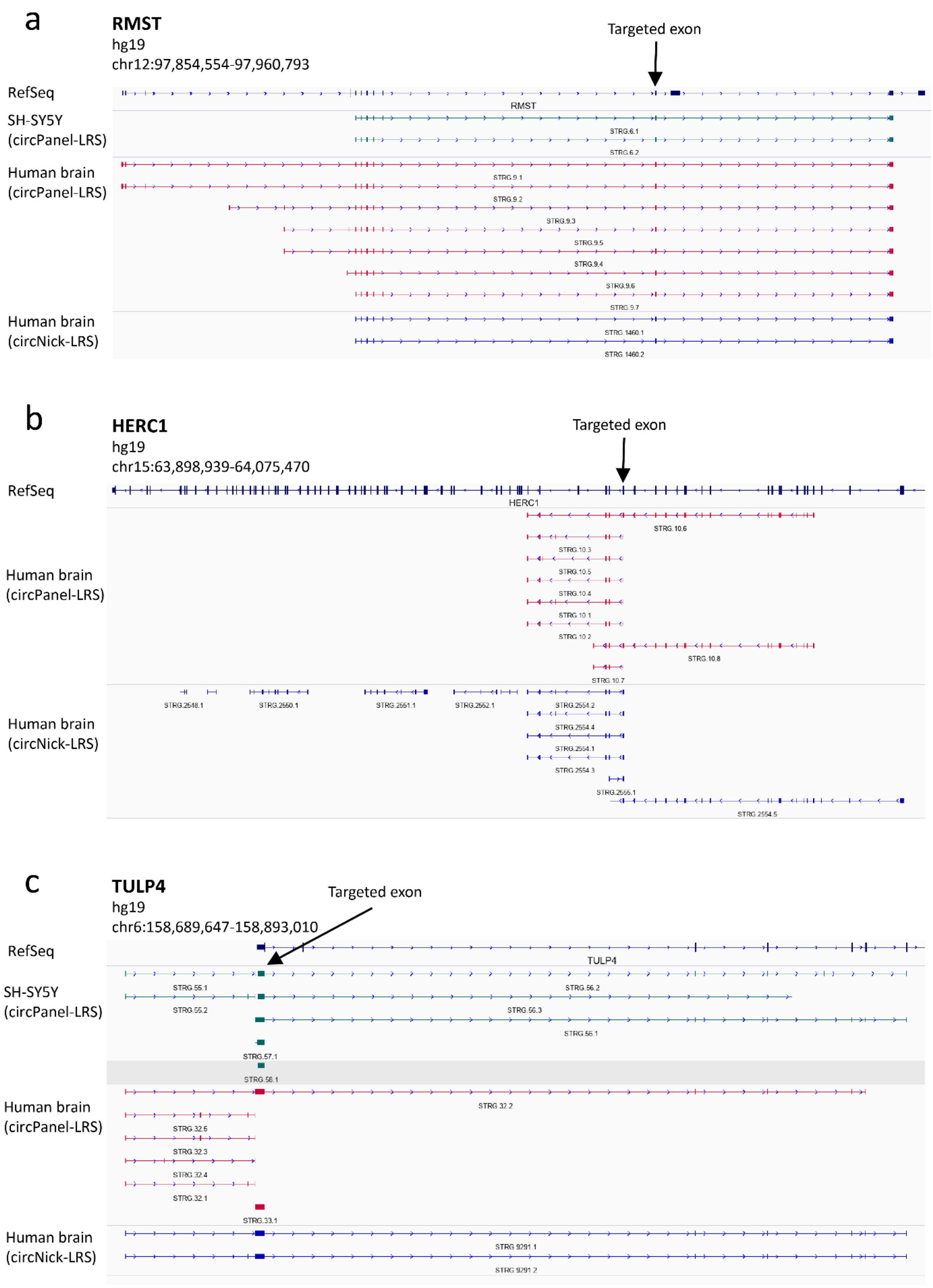

Supplementary Figure 5: circRNA isoforms detected by the panel approach.

Some of the circRNA isoforms that detected for circRMST **(a)**, circHERC1 **(b)** and circTULP4 **(c)** in human brain sample using circPanel-LRS. Targeted exons have been shown with black arrows.

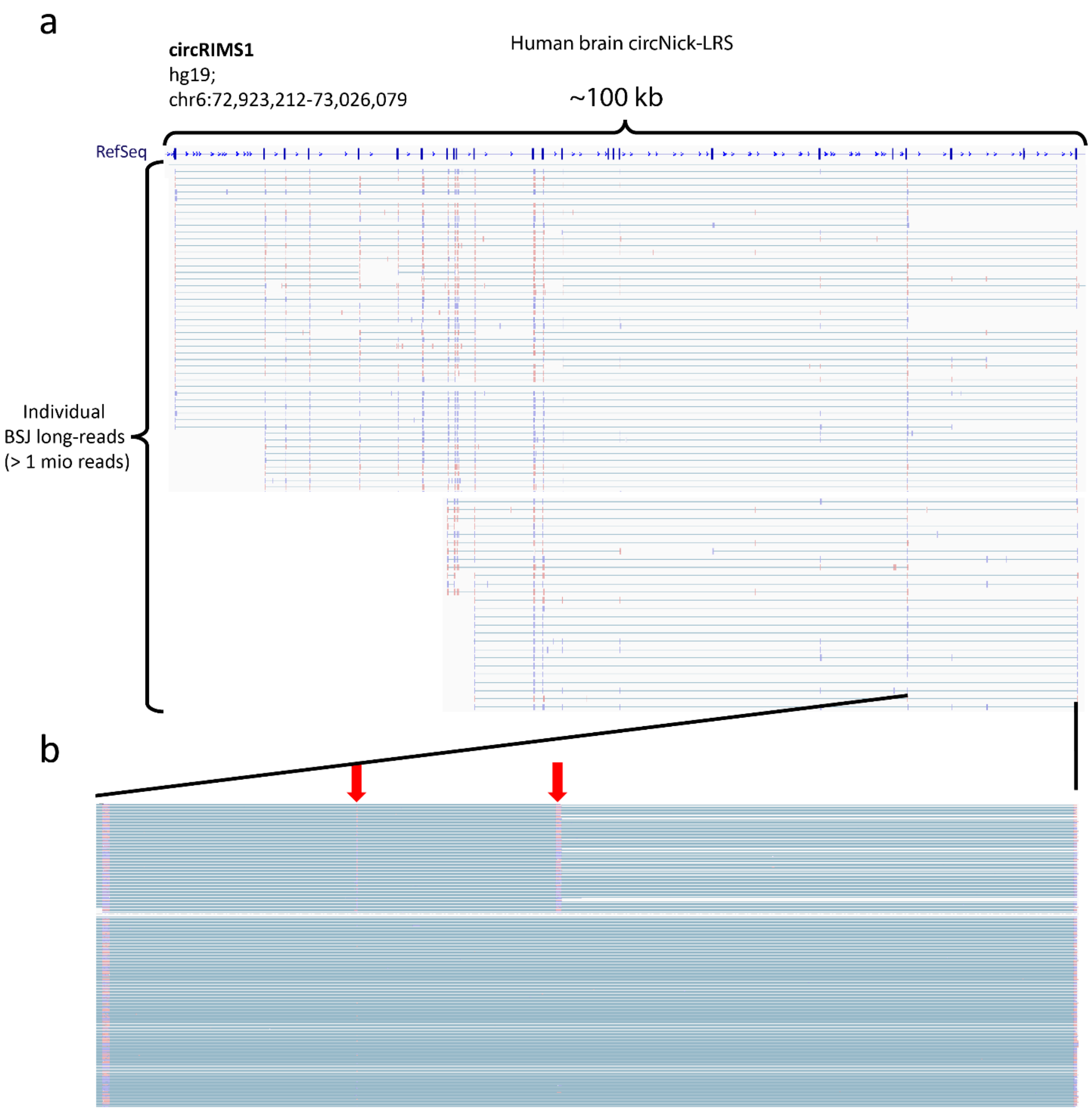

Supplementary Figure 6: Alternative exon usage in circRIMS1.

a) Sequencing reads of circRIMS1 in human brain sample using the circPanel-LRS strategy. **b)** Zooming in at an alternative exon usage in circRIMS1 (red arrows).

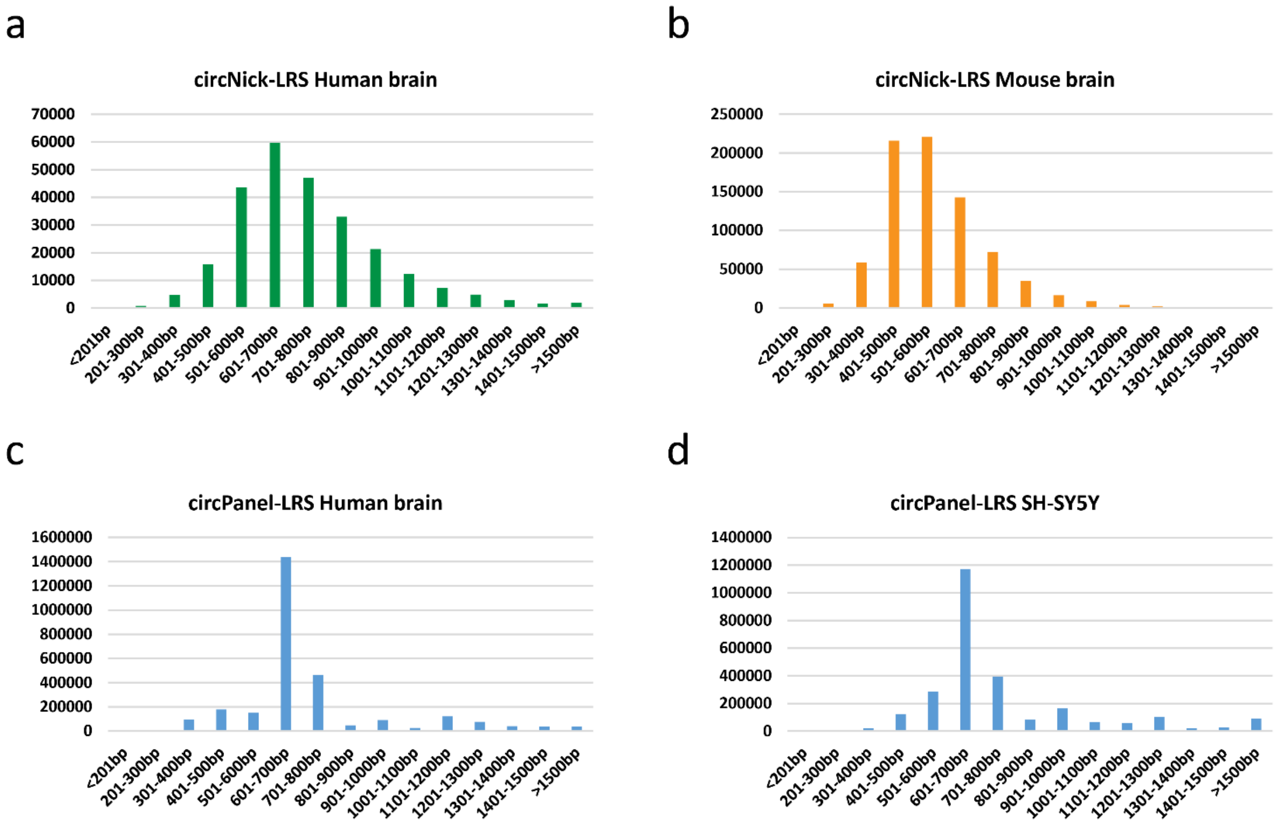

Supplementary Figure 7: Size distribution of full-length and BSJ-spanning reads.

Size distribution of full-length and BSJ-spanning reads in the circNick-LRS human brain dataset **(a)**, circNick-LRS mouse brain dataset **(b)**, circPanel-LRS human brain **(c)**, and circPanel-LRS SH-SY5Y neuroblastoma cell **(d)**.

Supplementary Table 1: Primers used for RT-PCR and Sanger sequencing

Primers that used for qPCR to evaluate circRNA enrichment efficiency		
**Target RNA transcript (mouse)**	**forward 5'>3'**	**reverse 5'>3'**
**βIII-tubulin**	GGGCTCCCAGGTTAAAGTCC	AAAATGGGGAGGACAGAGCC
**CircHipk3**	GGATCGGCCAGTCATGTATC	ACCGCTTGGCTCTACTTTGA
**18s rRNA**	CTCAACACGGGAAACCTCAC	AAATCGCTCCACCAACTAAGAA
**Primers that used for microexons RT-PCR validation and Sanger sequencing**		
**Target RNA transcript**	**forward 5'>3'**	**reverse 5'>3'**
**CircDTNA (human)**	GAACCTTTGCACCCCATGT	TGCATTAGTCAGCTTCTTAGCAGG
**CircDtna (mouse)**	GAACCTTTGCACCCCATGT	TGCATTCGTTAGCTTCTTAGCAGG
**CircEif4g3 (mouse)**	TTAACCATCTGCCCATGCCG	GGACGGATGGAAGGACTGCT
