# Supplementary material for: Nanopore sequencing of brain-derived full-length circRNAs reveals circRNA-specific exon usage, intron retention and microexons
Source: Nat Commun. 2021 Aug 10;12:4825. doi: 10.1038/s41467-021-24975-z (PMC8355340; doi:10.1038/s41467-021-24975-z)
Supplement: Supplementary file 20 — Reporting Summary [file 41467_2021_24975_MOESM20_ESM.pdf]

nature portfolio

Corresponding author(s): Jørgen Kjems; Karim Rahimi

Last updated by author(s): Jul 5, 2021

Reporting Summary

Nature Portfolio wishes to improve the reproducibility of the work that we publish. This form provides structure for consistency and transparency in reporting. For further information on Nature Portfolio policies, see our Editorial Policies and the Editorial Policy Checklist.

Statistics

For all statistical analyses, confirm that the following items are present in the figure legend, table legend, main text, or Methods section.

n/a	Confirmed	
☐	☒	The exact sample size (*n*) for each experimental group/condition, given as a discrete number and unit of measurement
☐	☒	A statement on whether measurements were taken from distinct samples or whether the same sample was measured repeatedly
☒	☐	The statistical test(s) used AND whether they are one- or two-sided<br>*Only common tests should be described solely by name; describe more complex techniques in the Methods section.*
☒	☐	A description of all covariates tested
☒	☐	A description of any assumptions or corrections, such as tests of normality and adjustment for multiple comparisons
☐	☒	A full description of the statistical parameters including central tendency (e.g. means) or other basic estimates (e.g. regression coefficient) AND variation (e.g. standard deviation) or associated estimates of uncertainty (e.g. confidence intervals)
☒	☐	For null hypothesis testing, the test statistic (e.g. *F*, *t*, *r*) with confidence intervals, effect sizes, degrees of freedom and *P* value noted<br>*Give P values as exact values whenever suitable.*
☒	☐	For Bayesian analysis, information on the choice of priors and Markov chain Monte Carlo settings
☒	☐	For hierarchical and complex designs, identification of the appropriate level for tests and full reporting of outcomes
☒	☐	Estimates of effect sizes (e.g. Cohen's *d*, Pearson's *r*), indicating how they were calculated

Our web collection on statistics for biologists contains articles on many of the points above.

Software and code

Policy information about availability of computer code

Data collection	Nanopore long-read data was collected using MinKNOW version 1.0.1 (Oxford Nanopore Technologies).<br>RNAseq short-read data was made using DNA nanoball sequencing technology and BGISEQ-500 sequencing platform.
Data analysis	Guppy (v 3.4.5), NanoFilt (v 2.6.0), Nanoplot (v 1.29.0), FLAIR (v 1.4), Trim Galore (v 0.4.1), CIRI2 (v 2.0.6), find_circ (v 1), FastQC v0.11.5, pblat v35, UCSC liftOver tool (2015-04-21), R version 3.5.1, Microsoft Excel 2016. Custom perl and bash scripts were used as outlined in Material and methods.<br>Custom perl and bash scripts can be found on github (https://github.com/omiics-dk/long_read_circRNA).<br>Short-read data circRNAs were quantified using CIRI2 (v 2.0.6) and find_circ (v 1) circRNA detection tools.

For manuscripts utilizing custom algorithms or software that are central to the research but not yet described in published literature, software must be made available to editors and reviewers. We strongly encourage code deposition in a community repository (e.g. GitHub). See the Nature Portfolio guidelines for submitting code & software for further information.

Data

Policy information about availability of data

All manuscripts must include a data availability statement. This statement should provide the following information, where applicable:

- Accession codes, unique identifiers, or web links for publicly available datasets
- A description of any restrictions on data availability
- For clinical datasets or third party data, please ensure that the statement adheres to our policy

The long-read and short-read sequencing data were deposited to the Gene Expression Omnibus (GEO) repository database with the accession number GSE127059.

The following circRNA databases were used: circBase (http://www.circbase.org/), CIRCpedia (https://www.picb.ac.cn/rnomics/circpedia/), and CircAtlas (http://circatlas.biols.ac.cn/)

Field-specific reporting

Please select the one below that is the best fit for your research. If you are not sure, read the appropriate sections before making your selection.

☒ Life sciences ☐ Behavioural & social sciences ☐ Ecological, evolutionary & environmental sciences

For a reference copy of the document with all sections, see nature.com/documents/nr-reporting-summary-flat.pdf

Life sciences study design

All studies must disclose on these points even when the disclosure is negative.

Sample size	One mouse sample and one human sample were used in the study. This is deemed sufficient to show successful circRNA detection in a proof-of-concept study.<br>For the circRNA panel sequencing one human brain sample and one sample of the SH-SY5Y cell line were used.
Data exclusions	No data points were excluded.
Replication	We sequenced the mouse and human brain samples using Oxford Nanopore MinION separately and detected thousands of circRNAs in both samples. More than 5,537 circRNAs were conserved between human and mouse. Replication of sequencing for each individual sample was not deemed necessary as this is a proof-of-concept study, and we wanted to show what was possible and not report on a specific disease and mechanism. However, we did compare results from nanopore sequencing to short read sequencing.<br>For panel sequencing one replicate of human brain and one replicate of the SH-SY5Y cell line were sequenced.
Randomization	Not relevant to our study, since only two samples were sequenced.
Blinding	Blinding was not relevant since the study has two groups with n=1.<br>There was no case-control setup where a non-blinded researcher could influence the outcome.

Reporting for specific materials, systems and methods

We require information from authors about some types of materials, experimental systems and methods used in many studies. Here, indicate whether each material, system or method listed is relevant to your study. If you are not sure if a list item applies to your research, read the appropriate section before selecting a response.

Materials & experimental systems

n/a	Involved in the study	
☒	☐	Antibodies
☐	☒	Eukaryotic cell lines
☒	☐	Palaeontology and archaeology
☐	☒	Animals and other organisms
☒	☐	Human research participants
☒	☐	Clinical data
☒	☐	Dual use research of concern

Methods

n/a	Involved in the study	
☒	☐	ChIP-seq
☒	☐	Flow cytometry
☒	☐	MRI-based neuroimaging

Eukaryotic cell lines

Policy information about cell lines

Cell line source(s)	We used the SH-SY5Y cell line that derives from human bone marrow.
Authentication	SH-SY5Y is a common cell line and did not need authentication.
Mycoplasma contamination	The cell line used was not tested for mycoplasma contamination.
Commonly misidentified lines<br>(See ICLAC register)	No commonly misidentified cell lines were used in this study.

Animals and other organisms

Policy information about studies involving animals; ARRIVE guidelines recommended for reporting animal research

Laboratory animals	For the preparation of mouse brain RNA, a two months old male mouse (strain 129S2/SV) was sacrificed and the entire brain was harvested and total RNA was obtained using Trizol. Mice had free access to food and water supplied ad libitum, and were kept under 14-hours light/10-hours dark cycle and standard conditions of ambient temperature (22°C±1°C) and humidity (50%±10%).
Wild animals	The study did not involve wild animals.
Field-collected samples	The study did not involve samples collected from the field.
Ethics oversight	Animals were treated according to the regulation of "The Animal Experiments Inspectorate", the legal authority under the "Ministry of Environment and Food of Denmark" (https://www.foedevarestyrelsen.dk/english/Animal/AnimalWelfare/The-Animal-Experiments-Inspectorate/Pages/default.aspx).

Note that full information on the approval of the study protocol must also be provided in the manuscript.
